# Supplementary material for: Pyridine Carboxamides Based on Sulfobetaines: Design, Reactivity, and Biological Activity
Source: Molecules. 2022 Nov 3;27(21):7542. doi: 10.3390/molecules27217542 (PMC9658115; doi:10.3390/molecules27217542)

Supplementary materials

Pyridine Carboxamides Based on Sulfobetaines: Design, Reactivity, and Biological Activity

Eugene P. Kramarova[1], Sophia S. Borisevich[2], Edward M. Khamitov[2], Alexander A. Korlyukov[1], Pavel V. Dorovatovskii[3], Tatiana A. Shmigol[1], Vladislav V. Skarga[1], Konstantin S. Mineev[4], Dmitry V. Tarasenko[1], Alexey A. Lagunin[5], Ivan A. Boldyrev[4], Yuri I. Baukov[1] and Vadim V. Negrebetsky[1]*

[1] Department of Medicinal Chemistry and Toxicology, Pirogov Russian National Research Medical University, 117997 Moscow, Russia.

[2] Laboratory of Physical Chemistry, Ufa Institute of Chemistry UFRS RAS, pr. Oktyabrya 71, 450054 Ufa, Russia.

[3] National Research Center "Kurchatov Institute", pl. Akad. Kurchatova, 1, 123098 Moscow, Russia.

[4] Shemyakin-Ovchinnikov Institute of Bioorganic Chemistry, Russian Academy of Sciences, 16/10 Miklukho-Maklaya Str., 117997 Moscow, Russia.

Corresponding author – Vadim V. Negrebetsky, negrebetsky1@rsmu.ru

A Table of Contents

Figure S1. The structures of transition states systems under study **2**

Figure S2. ^{2}H NMR spectrum of compound 15 (46.1 MHz, DMSO-d$_6$). **3**

Figure S3. MD simulation of compounds 5 (A) and 12 (B) The cycle is taken as a stationary part when analyzing the movement of substituents. **3**

Table S1. ^{1}H, ^{13}C NMR chemical shifts (theoretical calculations) **4**

Table S2. Crystallographic data for compounds 9–14 **11**

Figure S4 NMR (experimental part) **13**

Figure S5

Transition states

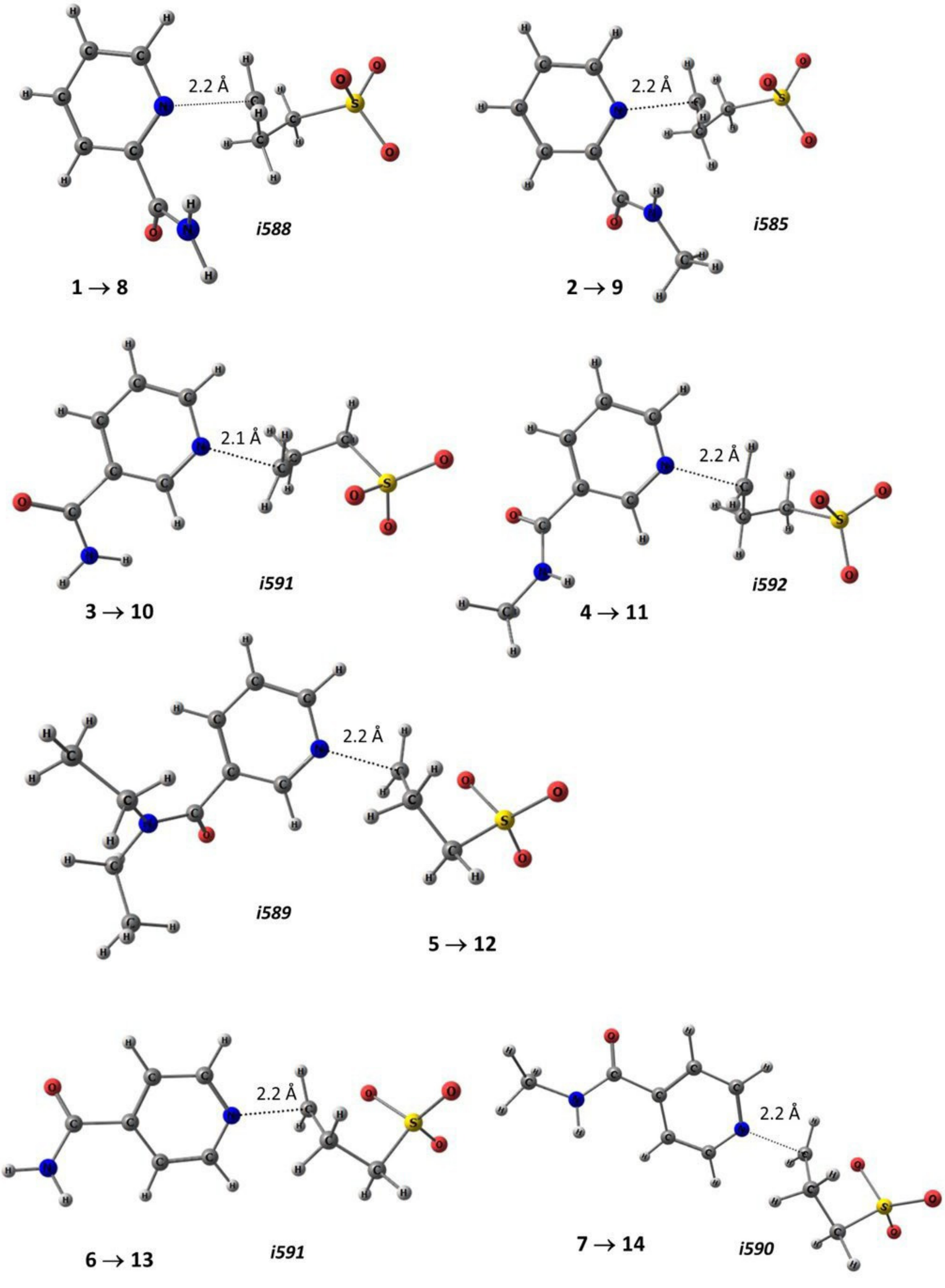

Figure S1. The structures of transition states systems under study.

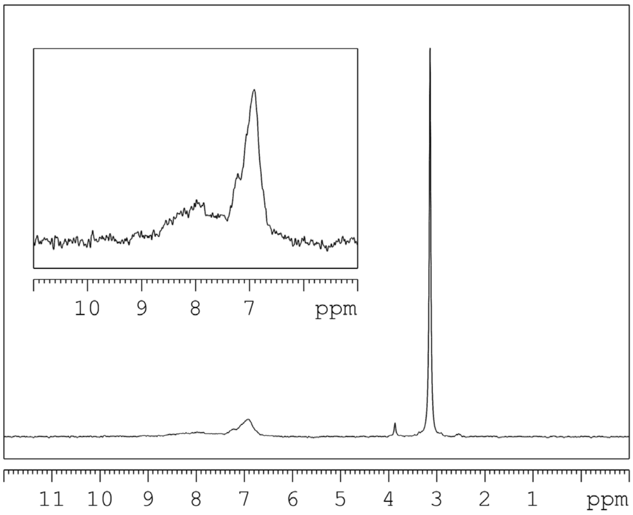

Figure S2. ^{2}H NMR spectrum of compound **15** (46.1 MHz, DMSO-d_6).

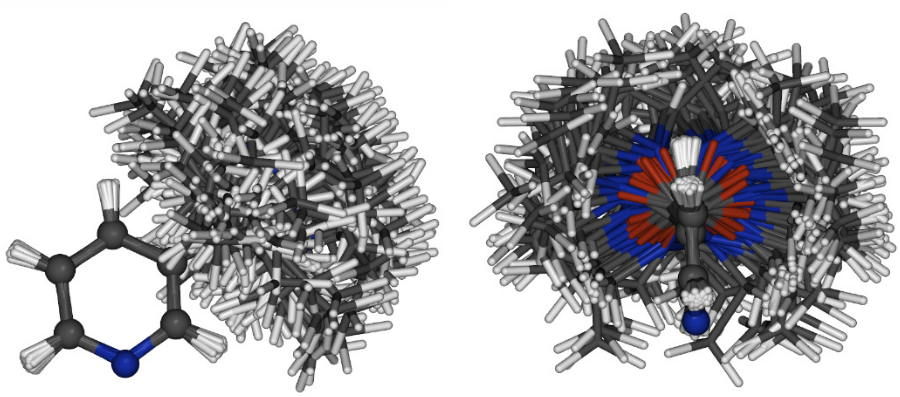

Compound 5 (A)

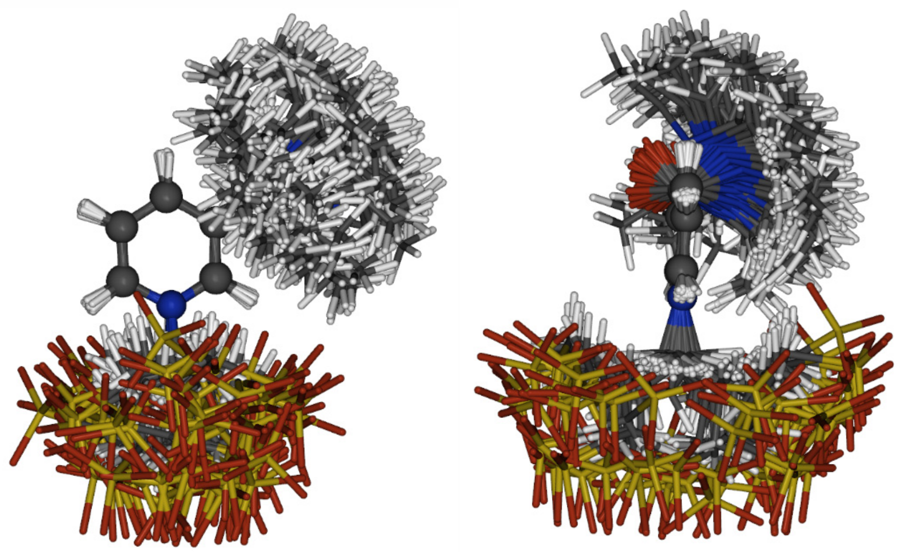

Compound 12 A

Figure S3. MD simulation of compounds 5 (A) and 12 (B) The cycle is taken as a stationary part when analyzing the movement of substituents.

Table S1. ^{1}H and ^{13}C NMR chemical shifts (theoretical calculations).

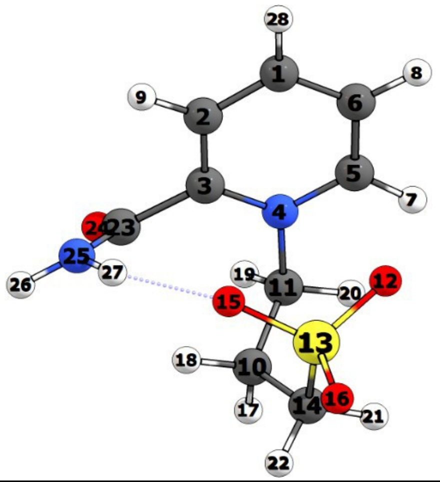

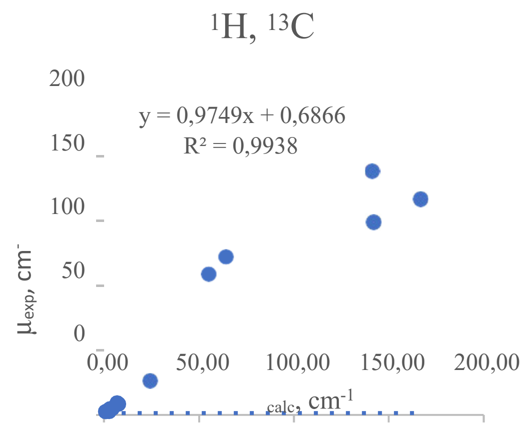

Compound 8					
№	atom	μ_{abs}, cm^{-1}	μ_{rev}, cm^{-1}	μ_{exp}, cm^{-1}	$\mu_{exp-rev}$, cm^{-1}
1	C	22.82	166.64	166.81	0.17
2	C	47.41	142.05	148.8	6.75
3	C	17.34	172.12		
4	N	21.94	0.00		
5	C	18.02	171.44		
6	C	48.83	140.63	126.63	-14.00
7	H	22.67	7.61		
8	H	23.35	6.93	9.07	2.14
9	H	23.20	7.08		
10	C	165.15	24.31	26.22	1.91
11	C	126.19	63.27	60.36	-2.91
12	O	44.69	0.00		
13	S	160.14	0.00		
14	C	135.24	54.22	47.1	-7.12
15	O	67.02	0.00		
16	O	91.03	0.00		
17	H	29.49	0.79	2.41	1.63
18	H	27.70	2.58	2.58	0.00
19	H	25.92	4.36	4.91	0.55
20	H	27.15	3.13	4.76	1.64
21	H	28.46	1.82	3.06	1.24
22	H	28.56	1.72	2.92	1.20
23	C	2.88	186.58		
24	O	-159.49	0.00		
25	N	128.11	0.00		
26	H	25.91	4.37		
27	H	22.66	7.62		
28	H	22.73	7.55	8.38	0.83

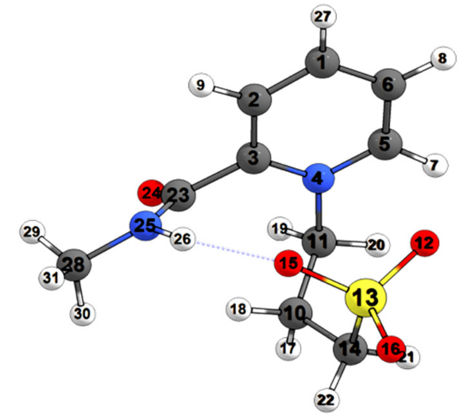

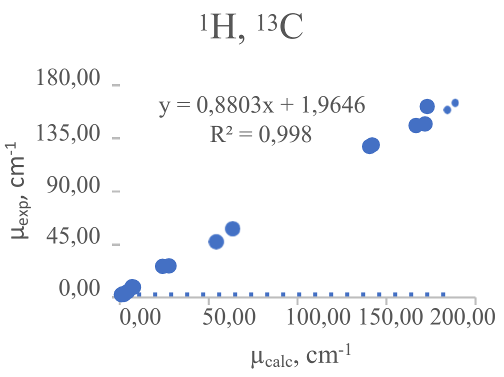

Compound 9					
№	atom	μ_{abs}, cm^{-1}	μ_{rev}, cm^{-1}	μ_{exp}, cm^{-1}	$\mu_{exp\text{-}rev}$, cm^{-1}
1	C	22.90	166.56	145.84	-20.72
2	C	47.58	141.88	129.53	-12.35
3	C	16.72	172.74	161.95	-10.79
4	N	21.57	0.00		
5	C	17.89	171.57	147.46	-24.11
6	C	49.05	140.41	127.95	-12.46
7	H	22.65	7.63	8.70	1.07
8	H	23.35	6.93	9.03	2.10
9	H	23.33	6.95	8.66	1.71
10	C	165.49	23.97	26.41	2.44
11	C	126.40	63.06	58.17	-4.89
12	O	43.53	0.00		
13	S	159.81	0.00		
14	C	135.35	54.11	47.43	-6.68
15	O	69.02	0.00		
16	O	89.89	0.00		
17	H	29.50	0.78	2.35	1.57
18	H	28.09	2.19	2.49	0.30
19	H	25.92	4.36	4.84	0.48
20	H	27.15	3.13		
21	H	28.47	1.81	3.03	1.22
22	H	28.59	1.69	2.97	1.28
23	C	3.38	186.08	162.09	-23.99
24	O	-147.90	0.00		
25	N	119.97	0.00		
26	H	22.85	7.43		
27	H	22.75	7.53	8.68	1.15
28	C	161.89	27.57	26.68	-0.89
29	H	27.69	2.59	3.00	0.41
30	H	28.22	2.06		
31	H	28.33	1.95		

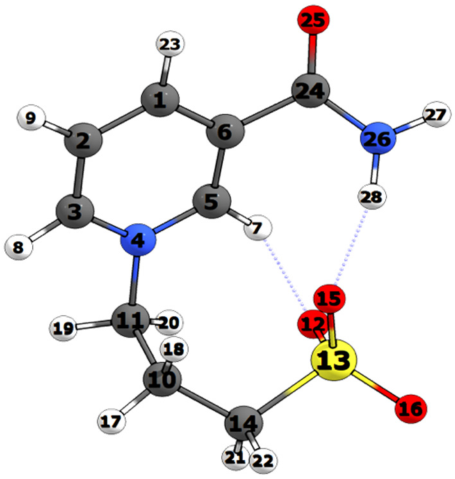

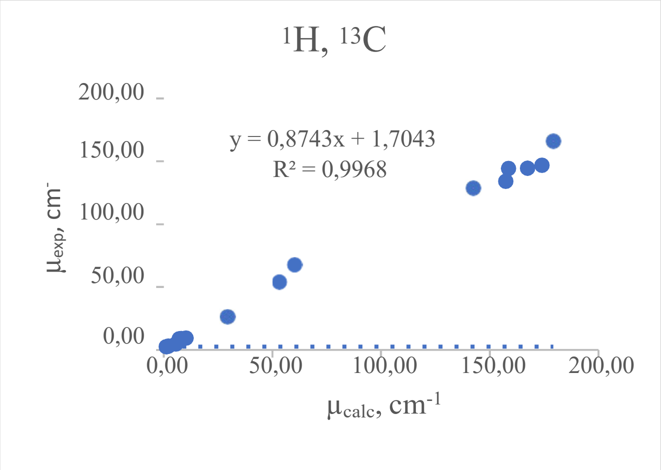

Compounds 10

№	atom	μ_{abs}, cm^{-1}	μ_{rev}, cm^{-1}	μ_{exp}, cm^{-1}	$\mu_{exp-rev}$, cm^{-1}
1	C	22.23	167.23	144.49	-22.74
2	C	47.10	142.36	128.55	-13.81
3	C	30.85	158.61	144.30	-14.31
4	N	3.38	0.00		
5	C	15.54	173.92	146.74	-27.18
6	C	32.12	157.34	134.11	-23.23
7	H	20.02	10.26	9.28	-0.98
8	H	23.13	7.15	8.82	1.67
9	H	23.30	6.98	8.12	1.14
10	C	160.18	29.28	26.08	-3.20
11	C	129.89	59.57	60.48	0.91
12	O	60.45	0.00		
13	S	149.45	0.00		
14	C	136.82	52.64	47.00	-5.64
15	O	60.89	0.00		
16	O	64.79	0.00		
17	H	28.88	1.40	2.46	1.06
18	H	29.20	1.08	2.36	1.28
19	H	27.21	3.07		
20	H	24.85	5.43	4.77	-0.66
21	H	28.23	2.05	2.93	0.88
22	H	28.43	1.85		
23	H	22.32	7.96	8.98	1.02
24	C	10.25	179.21	165.79	-13.42
25	O	-110.79	0.00		
26	N	131.23	0.00		
27	H	25.98	4.30		
28	H	22.22	8.06		

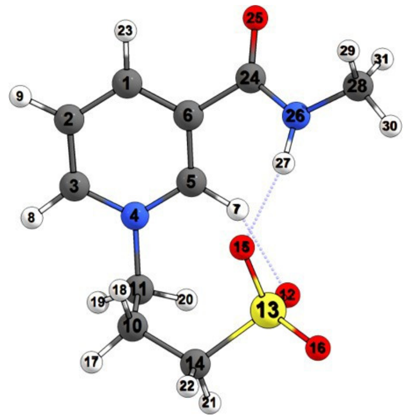

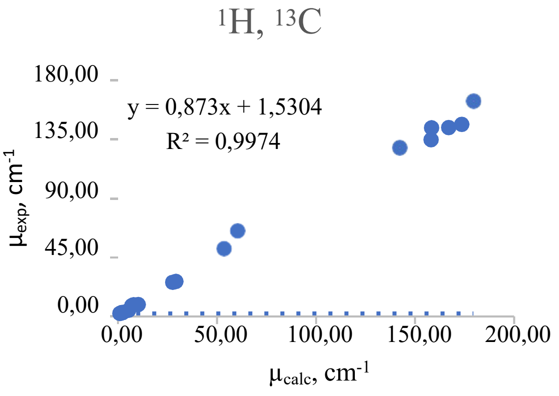

**Compounds 11**					
№	atom	μ_{abs}, cm^{-1}	μ_{rev}, cm^{-1}	μ_{exp}, cm^{-1}	$\mu_{exp-rev}$, cm^{-1}
1	C	22.46	167.00	144.07	-22.93
2	C	47.03	142.43	128.54	-13.89
3	C	31.32	158.14	134.69	-23.45
4	N	3.36	0.00		
5	C	15.93	173.53	146.45	-27.08
6	C	31.08	158.38	143.87	-14.51
7	H	20.06	10.22	9.22	-1.00
8	H	23.15	7.13		
9	H	23.30	6.98	8.11	1.13
10	C	160.22	29.24	26.61	-2.63
11	C	129.91	59.55	60.46	0.91
12	O	60.16	0.00		
13	S	149.16	0.00		
14	C	136.75	52.71	46.97	-5.74
15	O	62.73	0.00		
16	O	65.25	0.00		
17	H	28.89	1.39	2.48	1.09
18	H	29.19	1.09	2.32	1.23
19	H	27.22	3.06		
20	H	24.90	5.38	4.76	-0.62
21	H	28.23	2.05	2.96	0.91
22	H	28.43	1.85		
23	H	22.28	8.00	8.96	0.96
24	C	10.05	179.41	164.16	-15.25
25	O	-105.56	0.00		
26	N	122.74	0.00		
27	H	22.39	7.89		
28	C	161.70	27.76	26.03	-1.73
29	H	28.25	2.03	2.84	0.81
30	H	28.13	2.15	2.90	0.75
31	H	27.87	2.41	2.96	0.55

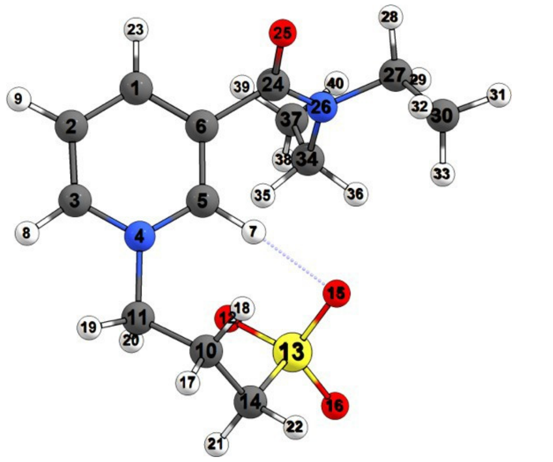

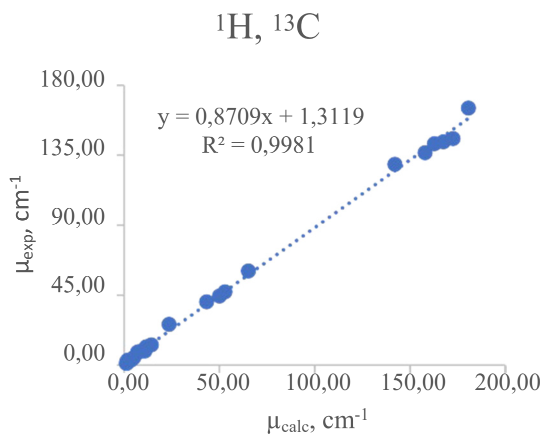

Compound 12

№	atom	μ_{abs}, cm^{-1}	μ_{rev}, cm^{-1}	μ_{exp}, cm^{-1}	$\mu_{exp-rev}$, cm^{-1}
1	C	22.06	167.40	143.55	-23.85
2	C	47.47	141.99	129.06	-12.93
3	C	31.57	157.89	136.49	-21.40
4	N	3.09			
5	C	17.00	172.45	145.61	-26.84
6	C	26.76	162.70	142.31	-20.39
7	H	19.39	10.89	9.04	-1.85
8	H	23.16	7.12	8.12	1.00
9	H	23.38	6.90	8.12	1.22
10	C	165.96	23.50	26.18	2.68
11	C	124.31	65.15	60.49	-4.66
12	O	80.35			
13	S	147.84			
14	C	136.72	52.74	47.00	-5.74
15	O	75.39			
16	O	71.82			
17	H	28.88	1.40	2.36	0.96
18	H	28.36	1.92	2.46	0.54
19	H	27.10	3.18		
20	H	25.29	4.99	4.76	-0.23
21	H	28.50	1.78	2.86	1.08
22	H	28.38	1.90	2.96	1.06
23	H	22.40	7.88	8.56	0.67
24	C	8.95	180.51	165.37	-15.14
25	O	-141.86			
26	N	90.79			
27	C	146.25	43.21	40.72	-2.49
28	H	26.82	3.46	3.23	-0.23
29	H	28.11	2.17		
30	C	177.94	11.52	11.76	0.24
31	H	30.11	0.17		
32	H	29.22	1.06	1.07	0.01

33	H	28.90	1.38	1.17	-0.21
34	C	139.52	49.94	44.42	-5.52
35	H	26.72	3.55	3.50	-0.05
36	H	27.40	2.88		
37	C	175.17	14.29	13.02	-1.27
38	H	29.85	0.43		
39	H	30.24	0.04		
40	H	29.51	0.77		

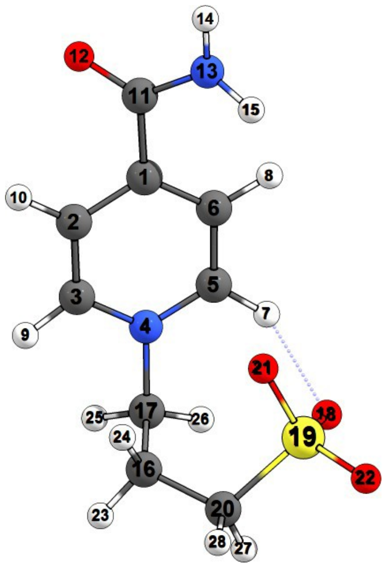

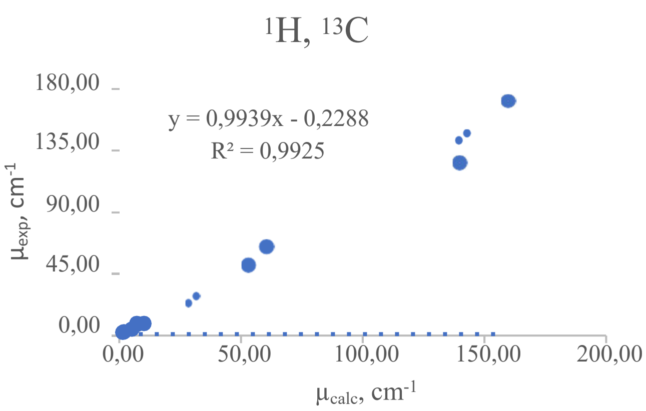

Compound 13

№	atom	μ_{abs}, cm^{-1}	μ_{rev}, cm^{-1}	μ_{exp}, cm^{-1}	$\mu_{exp-rev}$, cm^{-1}
1	C	19.12	170.34		
2	C	48.11	141.35	145.00	3.65
3	C	30.29	159.17	166.82	7.65
4	N	-0.43	0.00		
5	C	9.80	179.66		
6	C	49.50	139.96	126.00	-13.96
7	H	20.18	10.10	9.06	-1.04
8	H	23.10	7.18	8.38	1.20
9	H	23.05	7.23	9.06	1.83
10	H	23.10	7.18	8.38	1.20
11	C	8.45	181.01		
12	O	-145.76	0.00		
13	N	145.06	0.00		
14	H	25.98	4.30		
15	H	25.29	4.99		
16	C	159.19	30.27	26.22	-4.05
17	C	129.49	59.97	60.34	0.37
18	O	56.49	0.00		
19	S	152.70	0.00		
20	C	136.85	52.61	47.12	-5.49
21	O	42.29	0.00		
22	O	70.73	0.00		
23	H	28.90	1.38	2.49	1.11
24	H	29.08	1.20		
25	H	27.17	3.11		
26	H	25.12	5.16	4.85	-0.31
27	H	28.31	1.97	3.01	1.04
28	H	28.56	1.72		

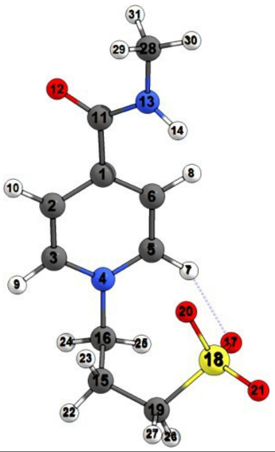

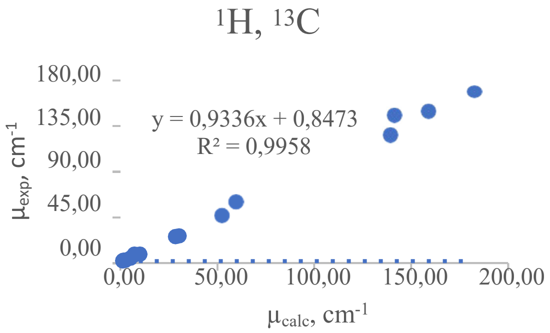

Compound 14					
№	atom	μ_{abs}, cm^{-1}	μ_{rev}, cm^{-1}	μ_{exp}, cm^{-1}	$\mu_{exp\text{-}rev}$, cm^{-1}
1	C	17.70	171.76		
2	C	47.92	141.54	145.64	4.10
3	C	30.38	159.08	149.09	-9.99
4	N	0.33	0.00		
5	C	10.09	179.37		
6	C	49.98	139.48	126.22	-13.26
7	H	20.22	10.06	8.96	-1.10
8	H	23.17	7.11	8.24	1.13
9	H	23.05	7.23	8.96	1.73
10	H	23.04	7.24	8.24	1.00
11	C	7.66	181.80	165.04	-16.76
12	O	-138.01	0.00		
13	N	136.32	0.00		
14	H	25.35	4.93		
15	C	159.22	30.24	26.64	-3.60
16	C	129.59	59.87	60.13	0.26
17	O	56.65	0.00		
18	S	152.67	0.00		
19	C	136.78	52.68	46.96	-5.72
20	O	43.63	0.00		
21	O	70.77	0.00		
22	H	28.91	1.37	2.44	1.07
23	H	29.08	1.20	2.33	1.13
24	H	27.17	3.11		
25	H	25.13	5.15	4.75	-0.40
26	H	28.32	1.96	2.95	0.99
27	H	28.56	1.72	2.83	1.11
28	C	160.97	28.49	26.10	-2.39
29	H	28.04	2.24		
30	H	28.29	1.99		
31	H	27.63	2.65	2.88	0.23

X-Ray Data

Table S2. Crystallographic data for compounds 9–14

	**9**	**10**	**11**	**12**	**13**	**14**
Brutto formula	$C_{10}H_{14}N_2O_4S$	$C_9H_{12}N_2O_4S$	$C_{20}H_{28}N_4O_8S_2$	$C_{13}H_{20}N_2O_4S$	$C_9H_{12}N_2O_4S$	$C_{10}H_{14}N_2O_4S$
Formula weight	258.29	244.27	516.58	300.37	244.27	258.29
Diffractometer	Bruker APEX-II CCD	MarExperts mardtb goniostat and Rayonix SX 165 detector	MarExperts mardtb goniostat and Rayonix SX 165 detector	Bruker D8 Quest	MarExperts mardtb goniostat and Rayonix SX 165 detector	Bruker APEX-II CCD
Scan mode	ω and ϕ scans	ϕ scan	ϕ scan	ϕ and ω scans	ϕ scan	ω and ϕ scans
Anode [Wavelength, Å]	MoKα [0.71073]	synchrotron [0.9699]	synchrotron [0.9699]	MoKα [0.71073]	synchrotron [0.80246]	MoKα [0.71073]
Crystal Dimensions, mm	0.21 × 0.23 × 0.28	0.12 × 0.2 × 0.3	0.02 × 0.02 × 0.4	0.04 × 0.2 × 0.2	0.11 × 0.3 × 0.3	0.15 × 0.28 × 0.35
Crystal color	colourless	colourless	colourless	colourless	colourless	colourless
Crystal system	triclinic	monoclinic	orthorhombic	triclinic	monoclinic	monoclinic
a, Å	6.8037(11)	13.146(3)	15.955(3)	7.688(2)	7.0983(14)	23.055(5)
b, Å	9.4524(15)	6.8693(14)	7.5251(15)	7.845(3)	7.5461(15)	7.6632(12)
c, Å	9.7051(15)	11.602(2)	18.825(4)	12.632(4)	19.322(4)	15.095(3)
α, °	112.531(3)	90	90	81.076(16)	90	90
β, °	93.787(4)	101.01(3)	90	77.240(15)	92.02(3)	116.413(5)
γ, °	97.361(4)	90	90	82.453(16)	90	90
Volume, $Å^3$	567.22(16)	1028.4(4)	2260.2(8)	730.3(4)	1034.3(4)	2388.6(7)
Density, gcm^{-3}	1.512	1.578	1.518	1.366	1.569	1.437
Temperature, K	120	100.15	100.15	120	120.0	120
T_{min}/T_{max}	0.6172/0.7461	0.74/0.91	0.43/0.85	0.4750/0.7461	0.876/0.953	0.6885/0.7461
μ, mm^{-1}	0.291	0.797	0.735	0.236	0.440	0.276
Space group	$P\bar{1}$	$P2_1/c$	Pbca	$P\bar{1}$	$P2_1/n$	C2/c
Z	2	4	4	2	4	8
F(000)	272	512	1088	320	512	1088
Reflections collected	5207	11035	10594	7492	10154	8955
Independent reflections	2207	2183	2397	2888	2264	3639
Reflections (I>2σ(I))	1662	1754	1643	1521	2105	3123
Parameters	155	146	156	184	146	155
R_{int}	0.0426	0.1409	0.1427	0.1196	0.0705	0.0203
$2\theta_{min}$ - $2\theta_{max}$, °	4.580 - 52.042	8.622 - 76.872	6.858 - 76.960	3.334 - 52.042	4.764 - 61.938	3.946 - 61.040
wR_2 (all reflections)	0.1592	0.1425	0.2516	0.2460	0.1151	0.0938
$R_1(I>\sigma(I))$	0.0555	0.0539	0.0831	0.0876	0.0421	0.0351
GOF	1.080	1.019	1.048	0.934	1.107	1.054
ρ_{min}/ρ_{max}, $eÅ^{-3}$	-0.608/0.410	-0.670/0.429	-0.891/0.829	-0.721/0.791	-0.487/0.402	-0.310/0.467

Figure S4 NMR (experimental part) Compound 1 HSQCMBC

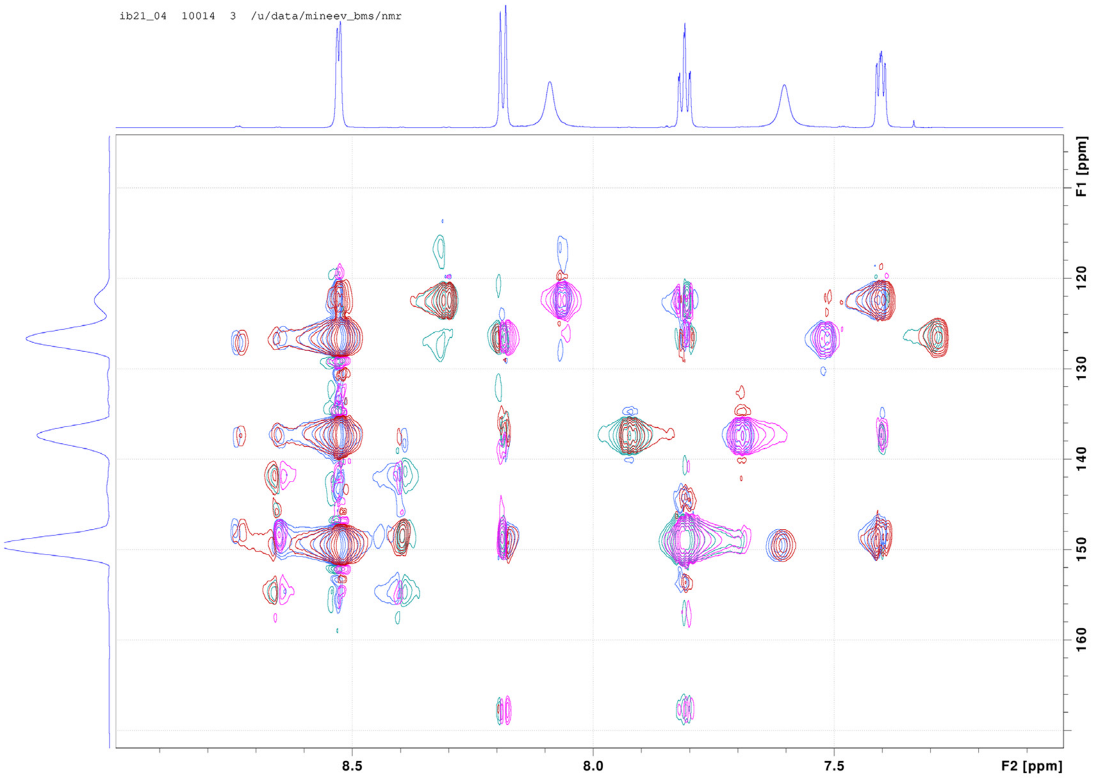

Compound 2 HSQCMBC

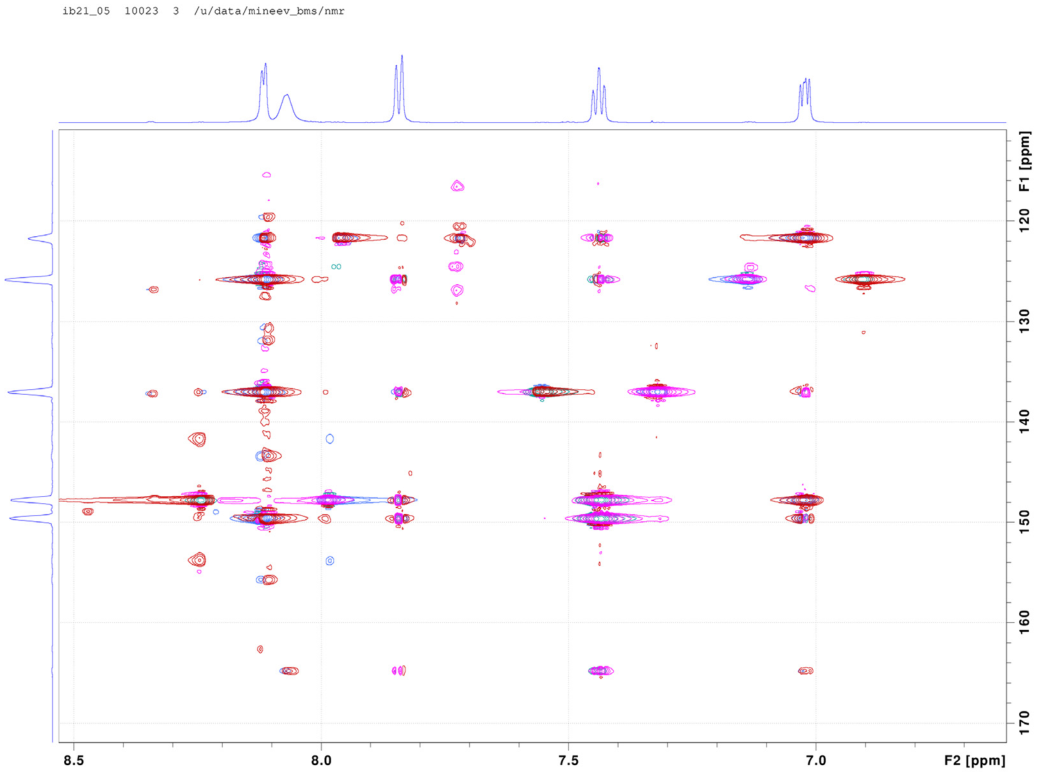

Compound 1 NOESY

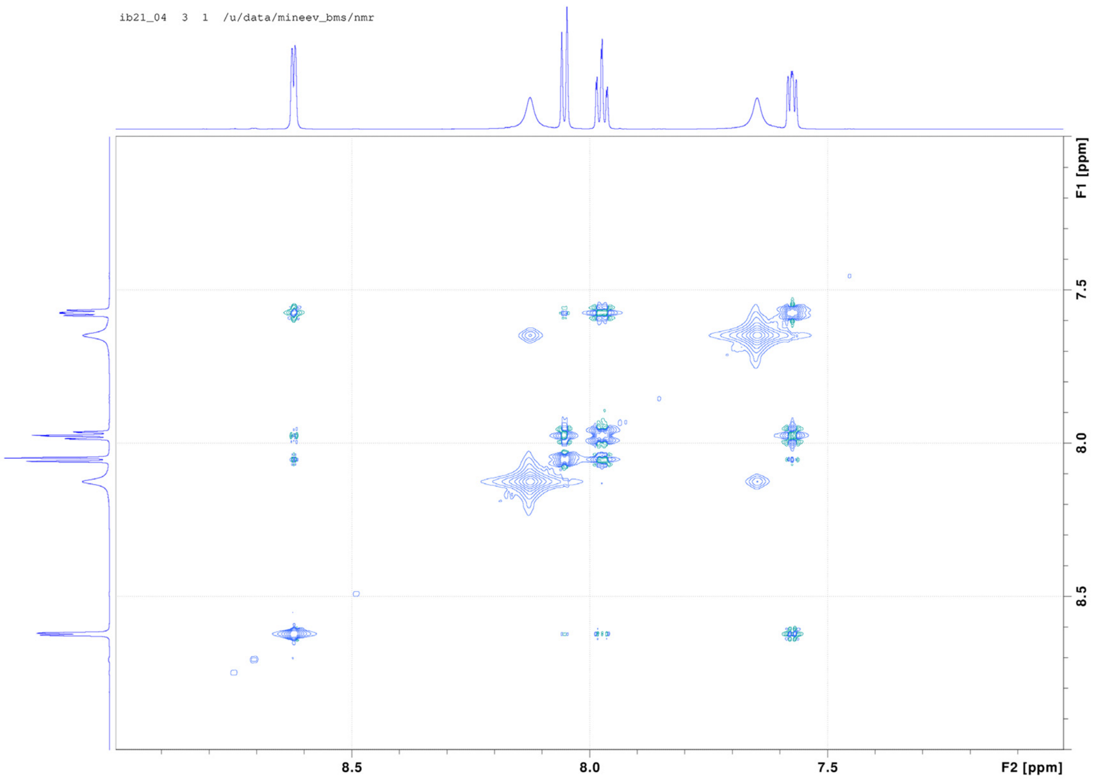

Compound 1 ROESY

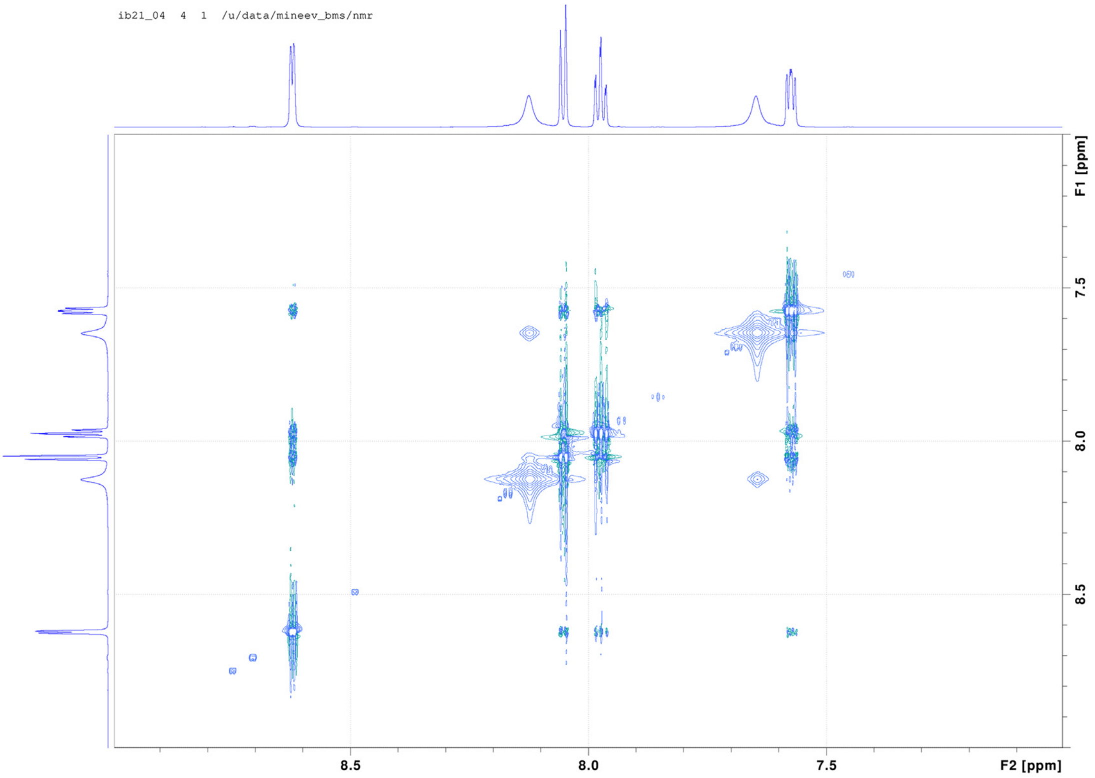

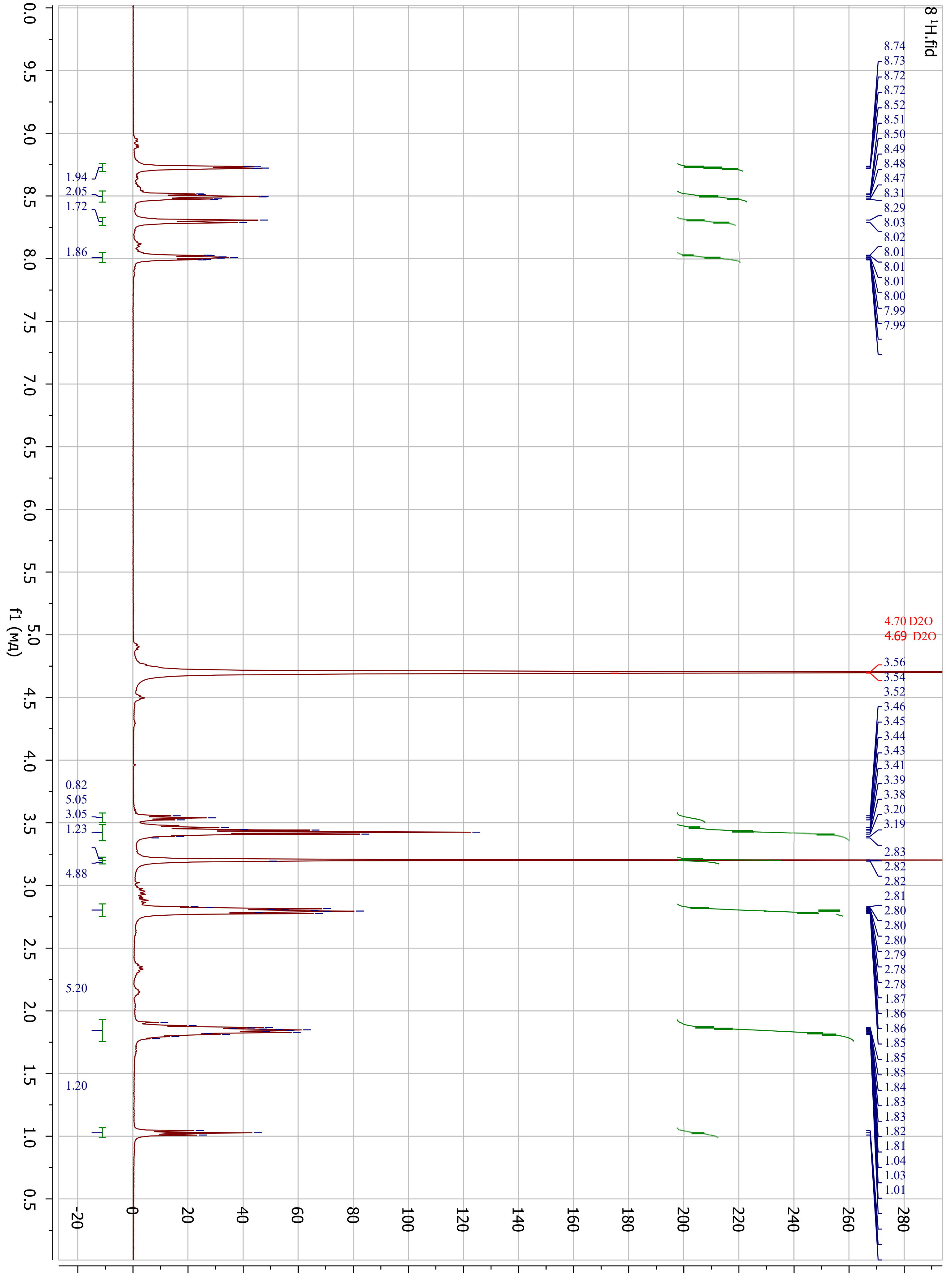
8 1H.fid
8.74
8.73
8.72
8.72
8.52
8.51
8.50
8.49
8.48
8.47
8.31
8.29
8.03
8.02
8.01
8.01
8.01
8.00
7.99
7.99
4.70 D2O
4.69 D2O
3.56
3.54
3.52
3.46
3.45
3.44
3.43
3.41
3.39
3.38
3.20
3.19
2.83
2.82
2.82
2.81
2.80
2.80
2.80
2.79
2.78
2.78
1.87
1.86
1.86
1.85
1.85
1.85
1.84
1.83
1.83
1.82
1.81
1.04
1.03
1.01
1.94
2.05
1.72
1.86
0.82
5.05
3.05
1.23
4.88
5.20
1.20
f1 (мд)

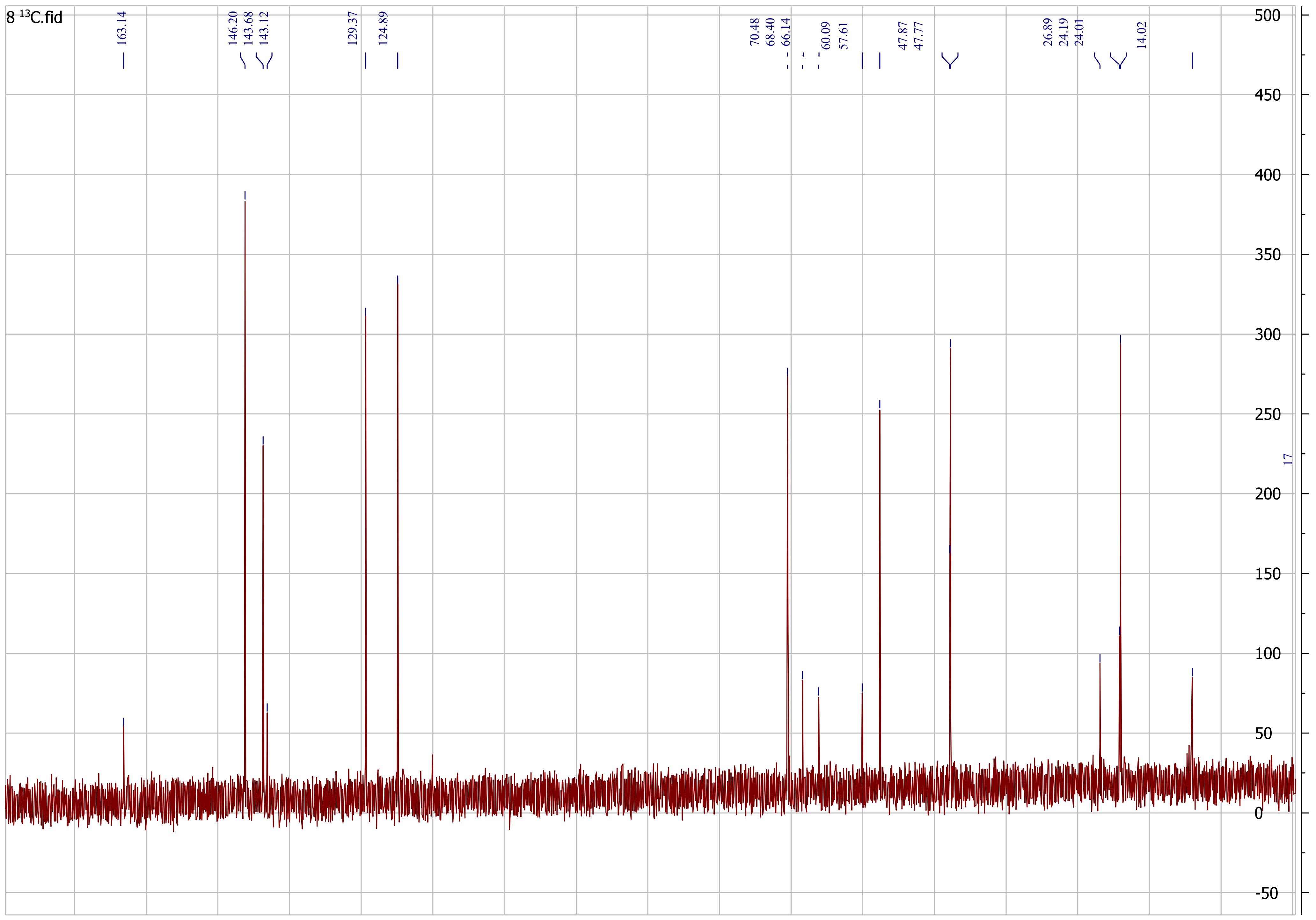
8 ^{13}C.fid
163.14
146.20
143.68
143.12
129.37
124.89
70.48
68.40
66.14
60.09
57.61
47.87
47.77
26.89
24.19
24.01
14.02
17
500
450
400
350
300
250
200
150
100
50
0
-50

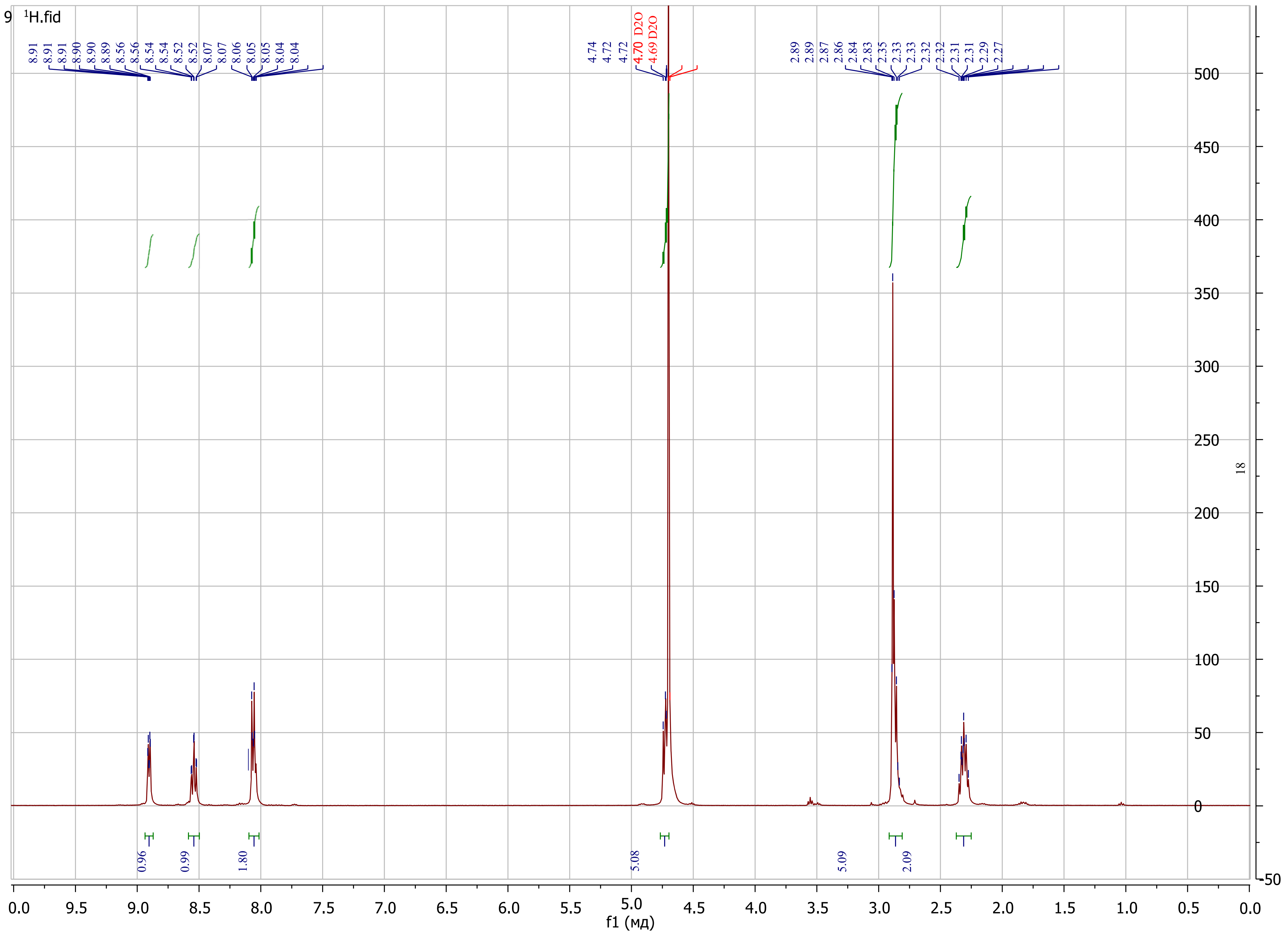
9 ¹H.fid
8.91 8.91 8.91 8.90 8.90 8.89 8.56 8.56 8.54 8.54 8.52 8.52 8.07 8.07 8.06 8.05 8.05 8.04 8.04
4.74 4.72 4.72 4.70 D2O 4.69 D2O
2.89 2.89 2.87 2.86 2.84 2.83 2.35 2.33 2.33 2.32 2.32 2.31 2.31 2.29 2.27
0.96
0.99
1.80
5.08
5.09
2.09
f1 (мд)

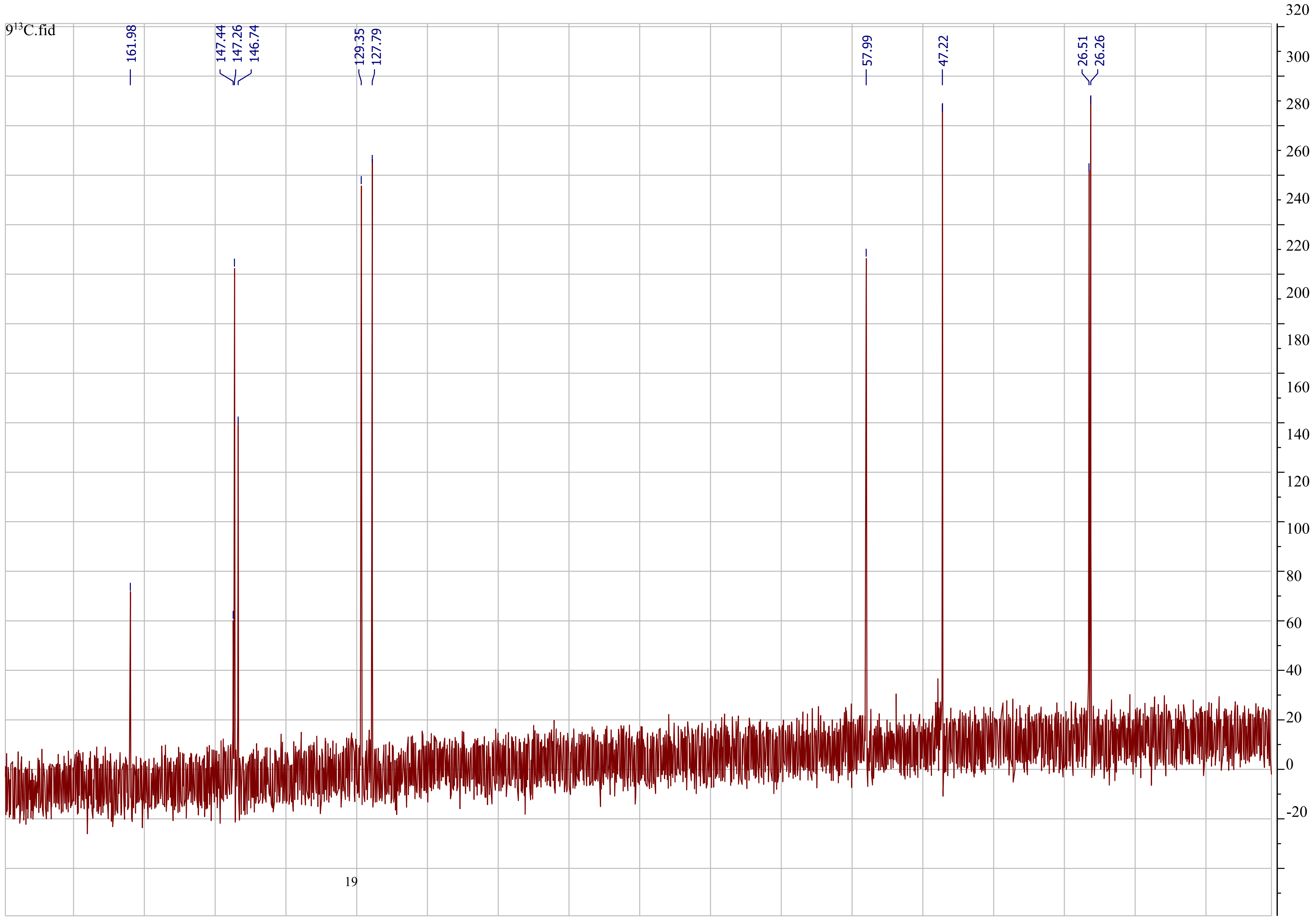
9 13C.fid
161.98
147.44
147.26
146.74
129.35
127.79
57.99
47.22
26.51
26.26
320
300
280
260
240
220
200
180
160
140
120
100
80
60
40
20
0
-20

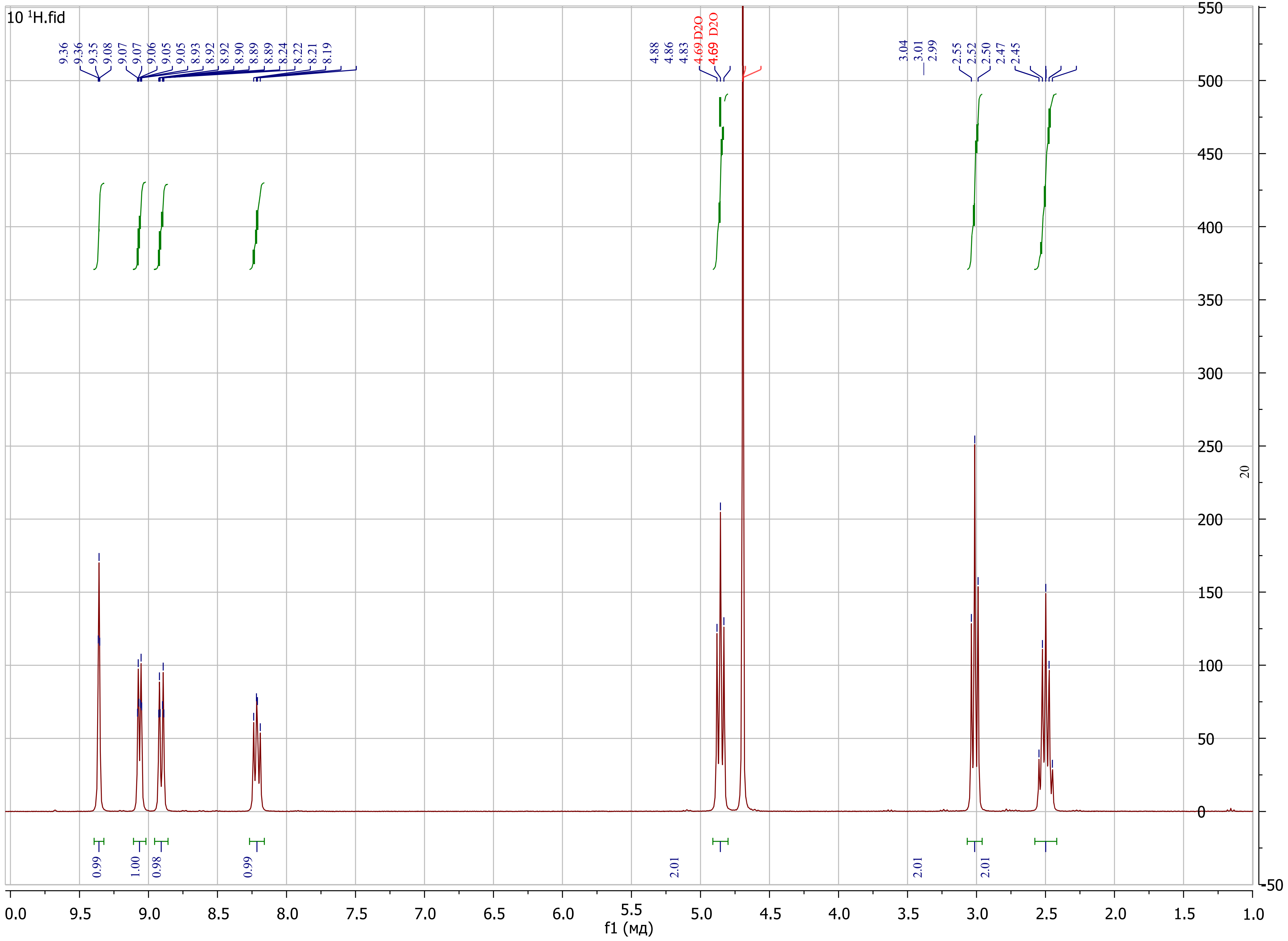
10 1H.fid
9.36
9.36
9.35
9.08
9.07
9.07
9.06
9.05
9.05
8.93
8.92
8.92
8.90
8.89
8.89
8.24
8.22
8.21
8.19
4.88
4.86
4.83
4.69 D2O
4.69 D2O
3.04
3.01
2.99
2.55
2.52
2.50
2.47
2.45
0.99
1.00
0.98
0.99
2.01
2.01
2.01
f1 (мд)

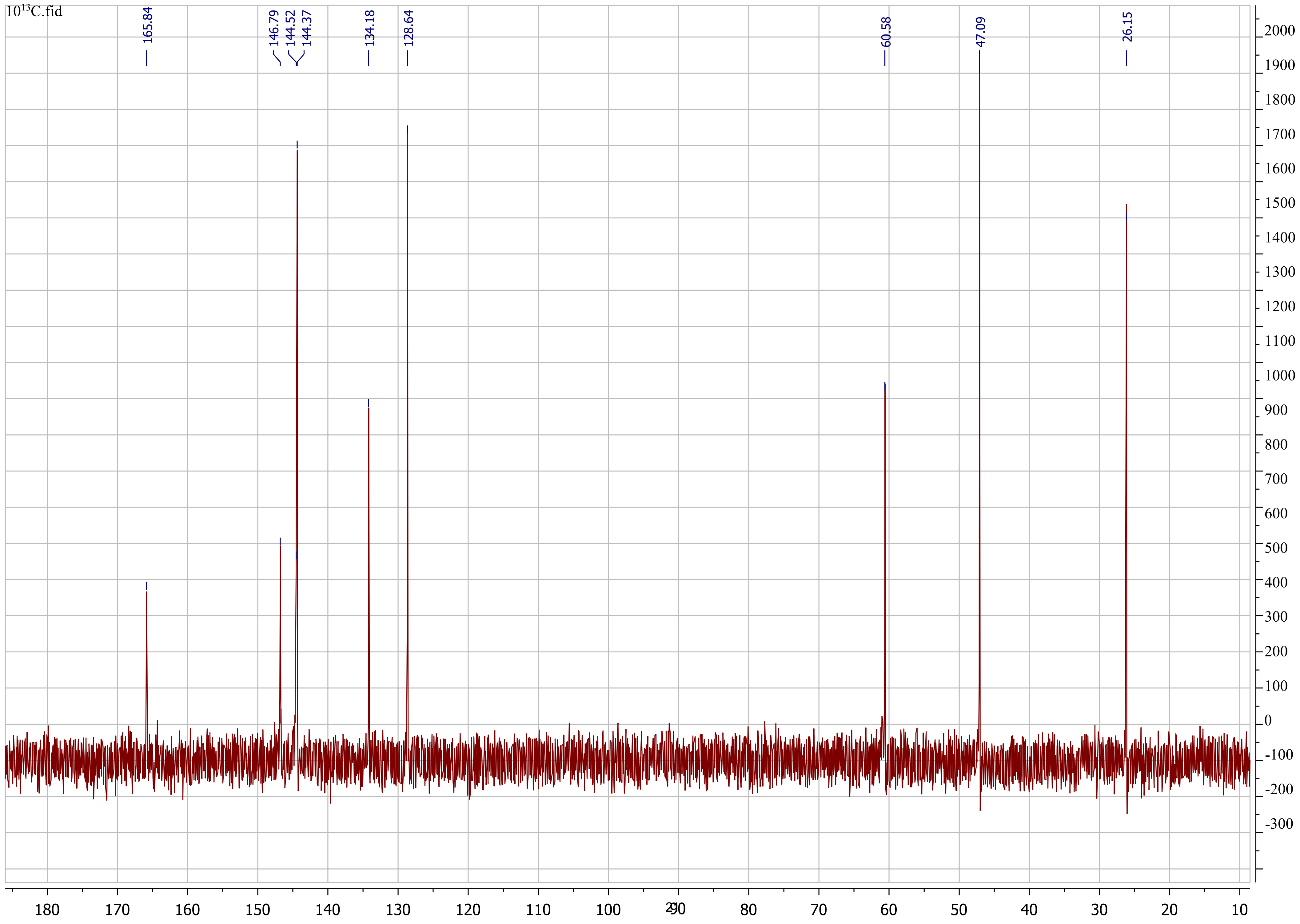
10[13]C.fid
165.84
146.79
144.52
144.37
134.18
128.64
60.58
47.09
26.15
2000
1900
1800
1700
1600
1500
1400
1300
1200
1100
1000
900
800
700
600
500
400
300
200
100
0
-100
-200
-300
180
170
160
150
140
130
120
110
100
90
80
70
60
50
40
30
20
10
22

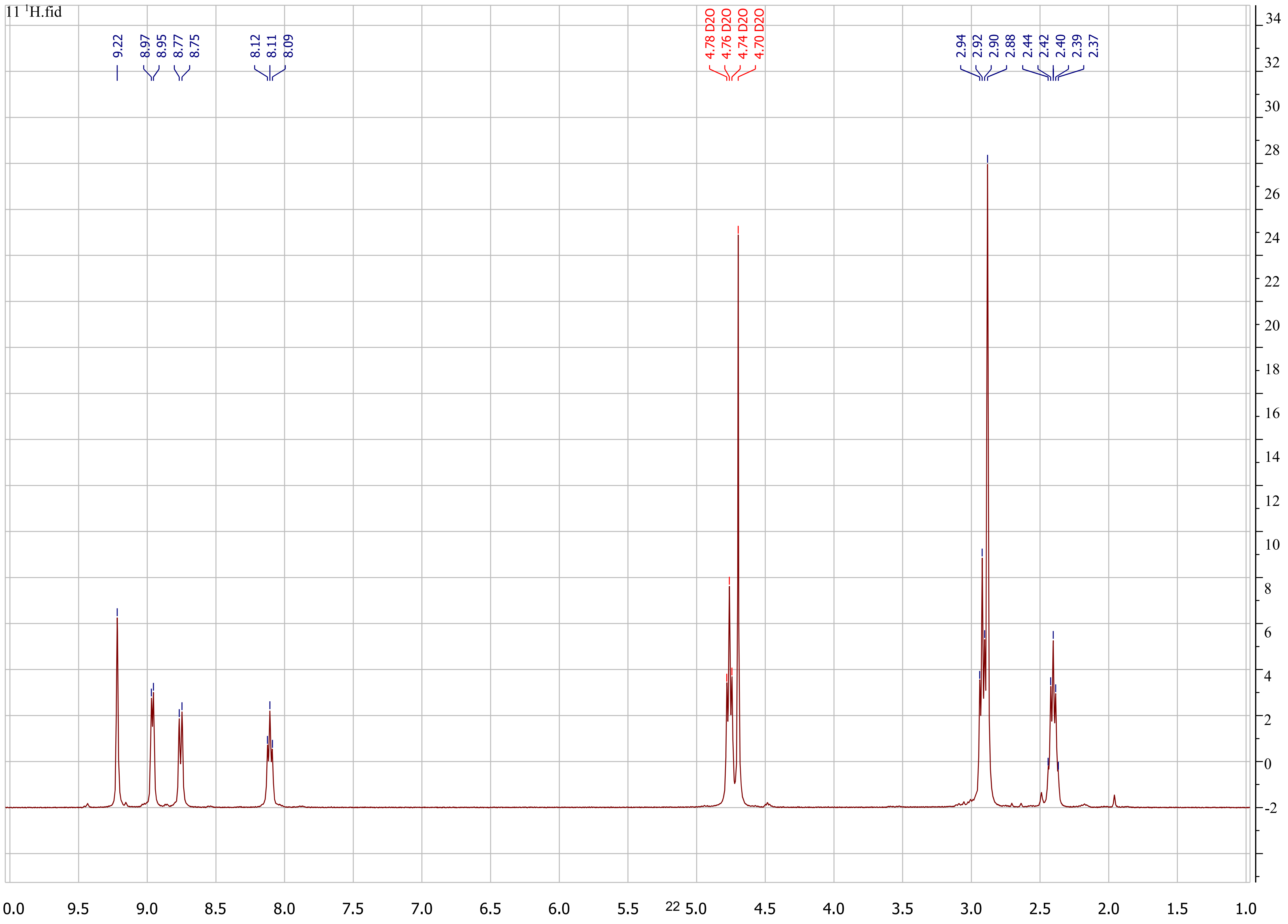

11 1H.fid
9.22
8.97
8.95
8.77
8.75
8.12
8.11
8.09
4.78 D2O
4.76 D2O
4.74 D2O
4.70 D2O
2.94
2.92
2.90
2.88
2.44
2.42
2.40
2.39
2.37

11 ^{13}C.fid

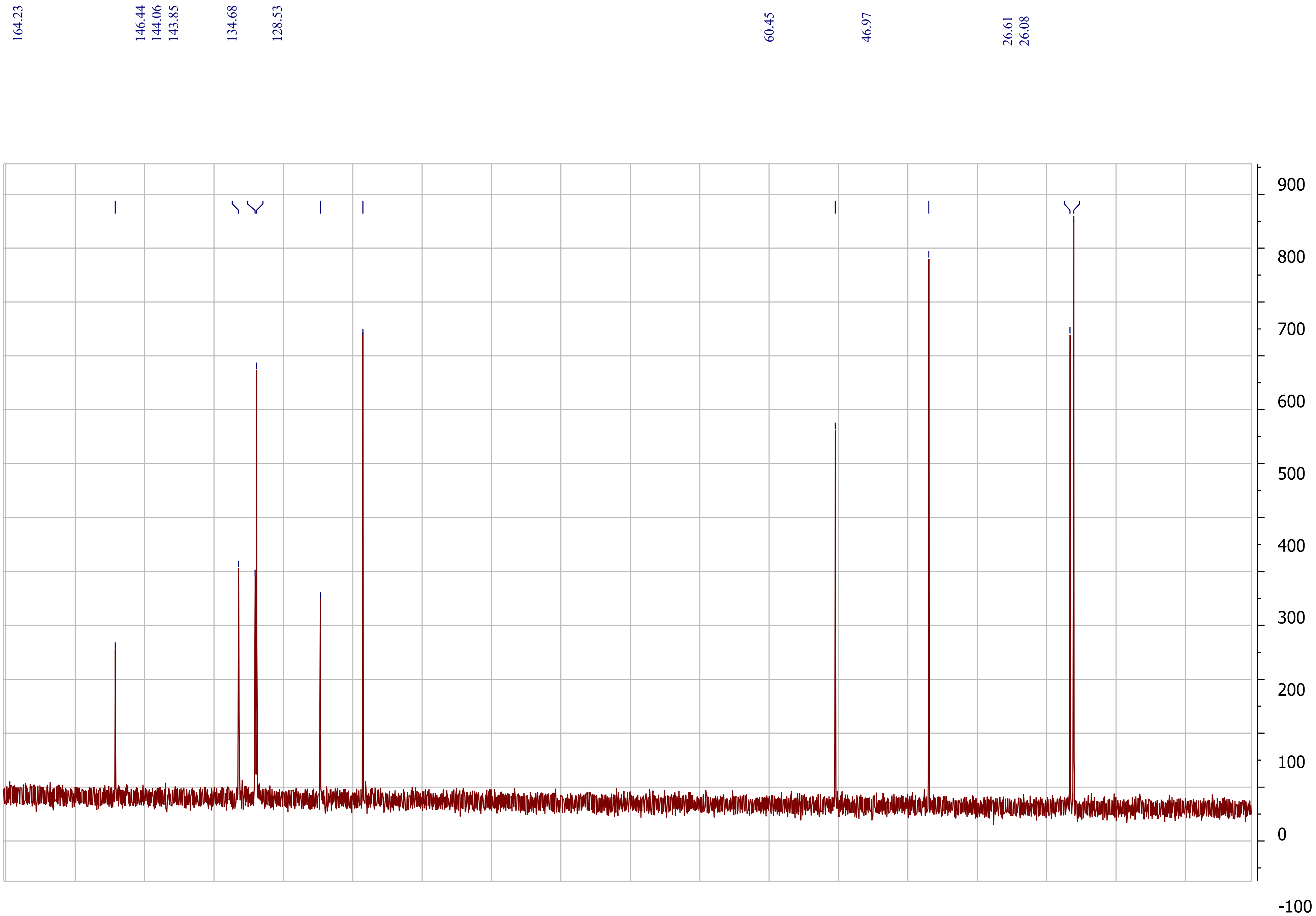
164.23
146.44
144.06
143.85
134.68
128.53
60.45
46.97
26.61
26.08
900
800
700
600
500
400
300
200
100
0
-100

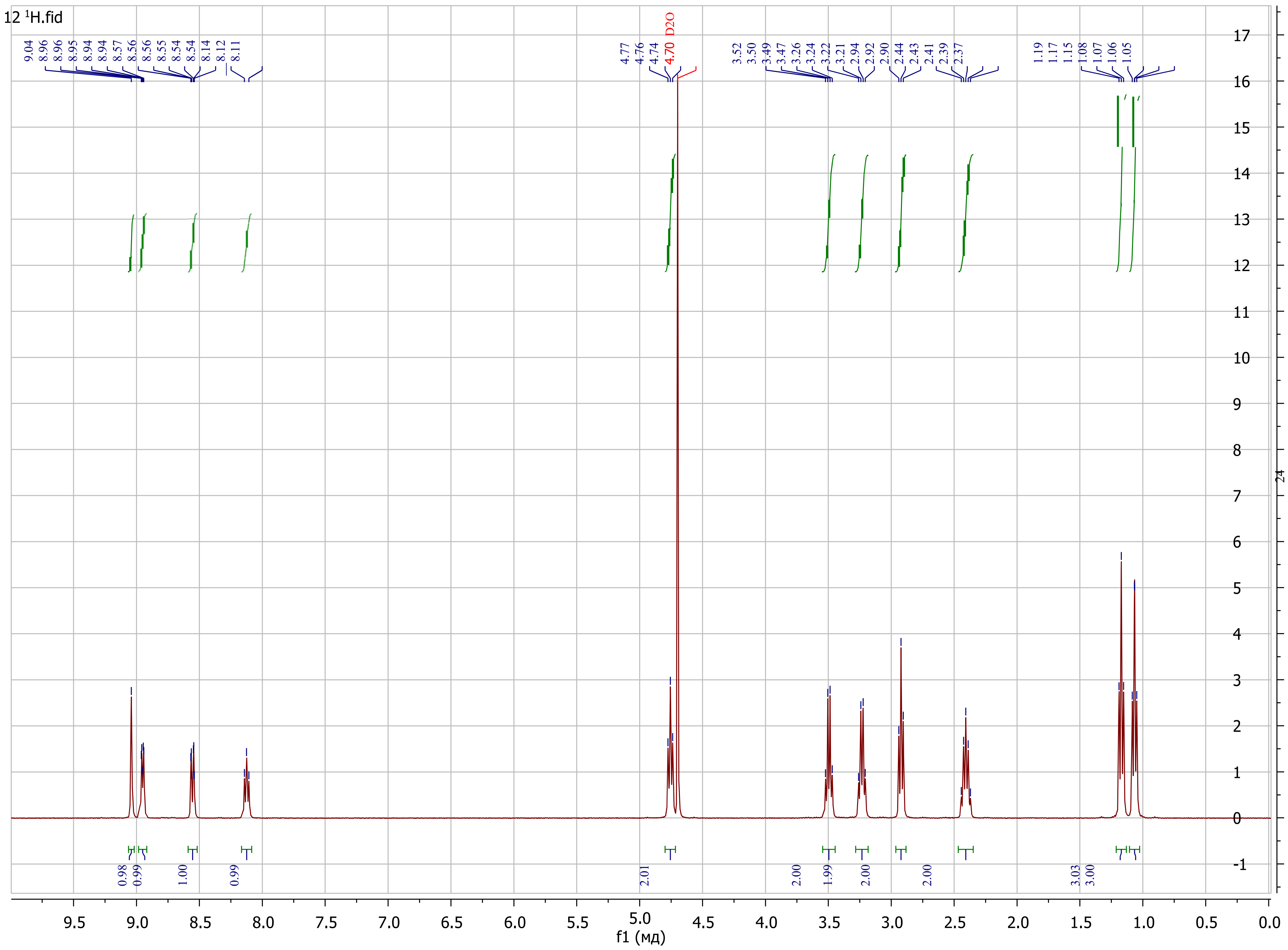
12 1H.fid
9.04 8.96 8.96 8.95 8.94 8.94 8.57 8.56 8.56 8.55 8.54 8.54 8.14 8.12 8.11
4.77 4.76 4.74 4.70 D2O
3.52 3.50 3.49 3.47 3.26 3.24 3.22 3.21 2.94 2.92 2.90 2.44 2.43 2.41 2.39 2.37
1.19 1.17 1.15 1.08 1.07 1.06 1.05
0.98 0.99 1.00 0.99 2.01 2.00 1.99 2.00 2.00 3.03 3.00
f1 (мд)

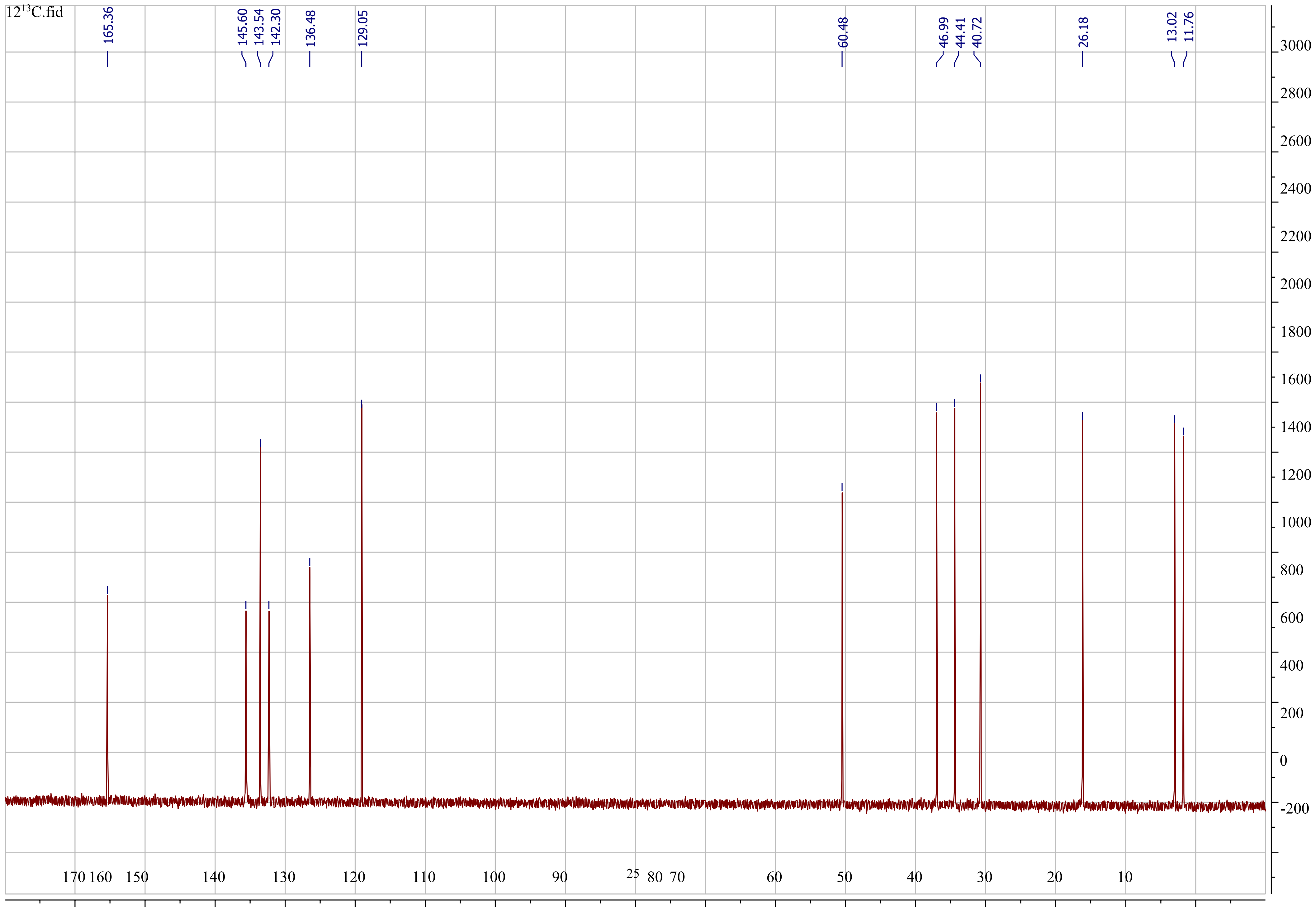

12 13C.fid
165.36
145.60
143.54
142.30
136.48
129.05
60.48
46.99
44.41
40.72
26.18
13.02
11.76
3000
2800
2600
2400
2200
2000
1800
1600
1400
1200
1000
800
600
400
200
0
-200
170 160 150
140
130
120
110
100
90
25 80 70
60
50
40
30
20
10

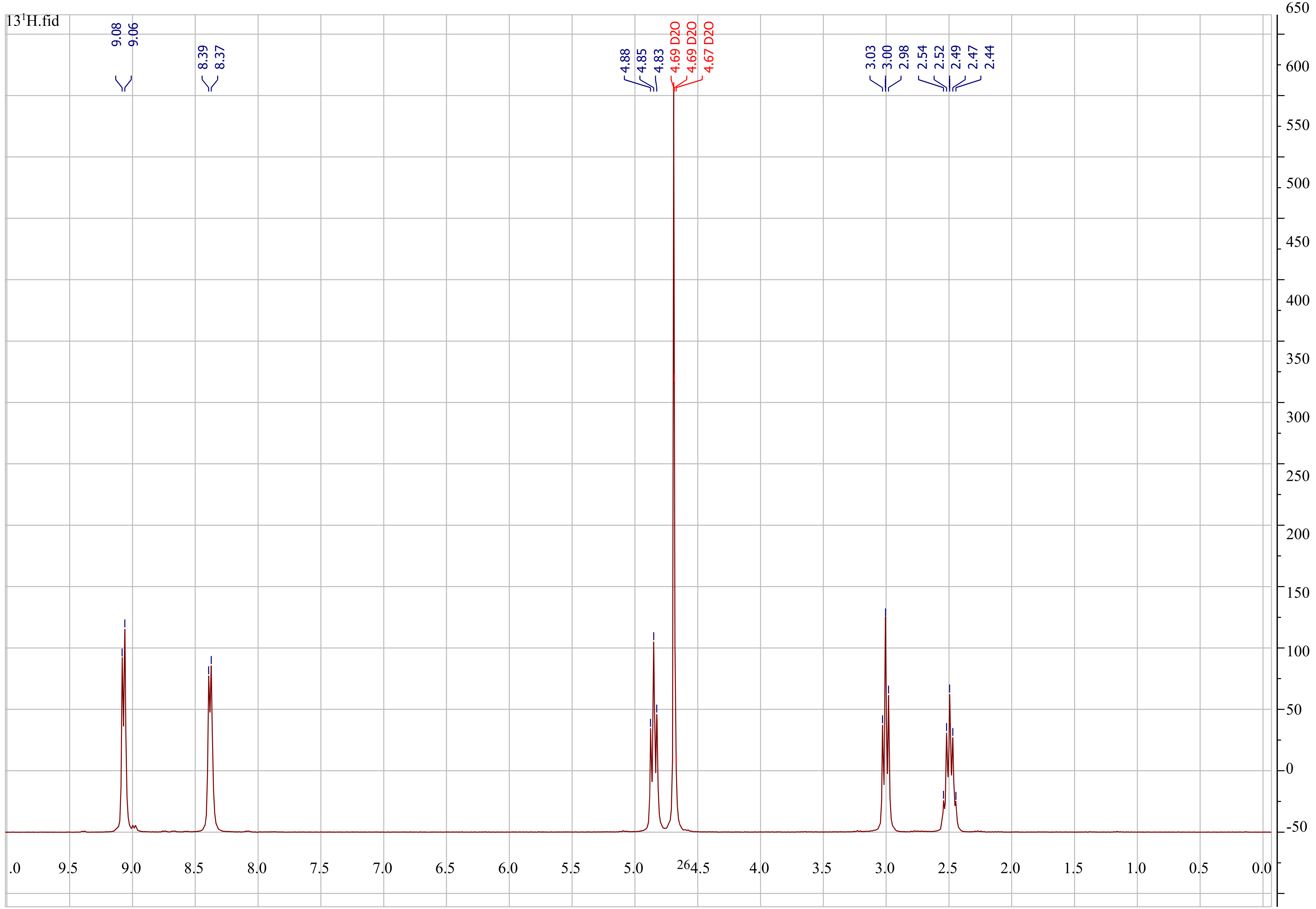

13¹H.fid
9.08
9.06
8.39
8.37
4.88
4.85
4.83
4.69 D2O
4.69 D2O
4.67 D2O
3.03
3.00
2.98
2.54
2.52
2.49
2.47
2.44

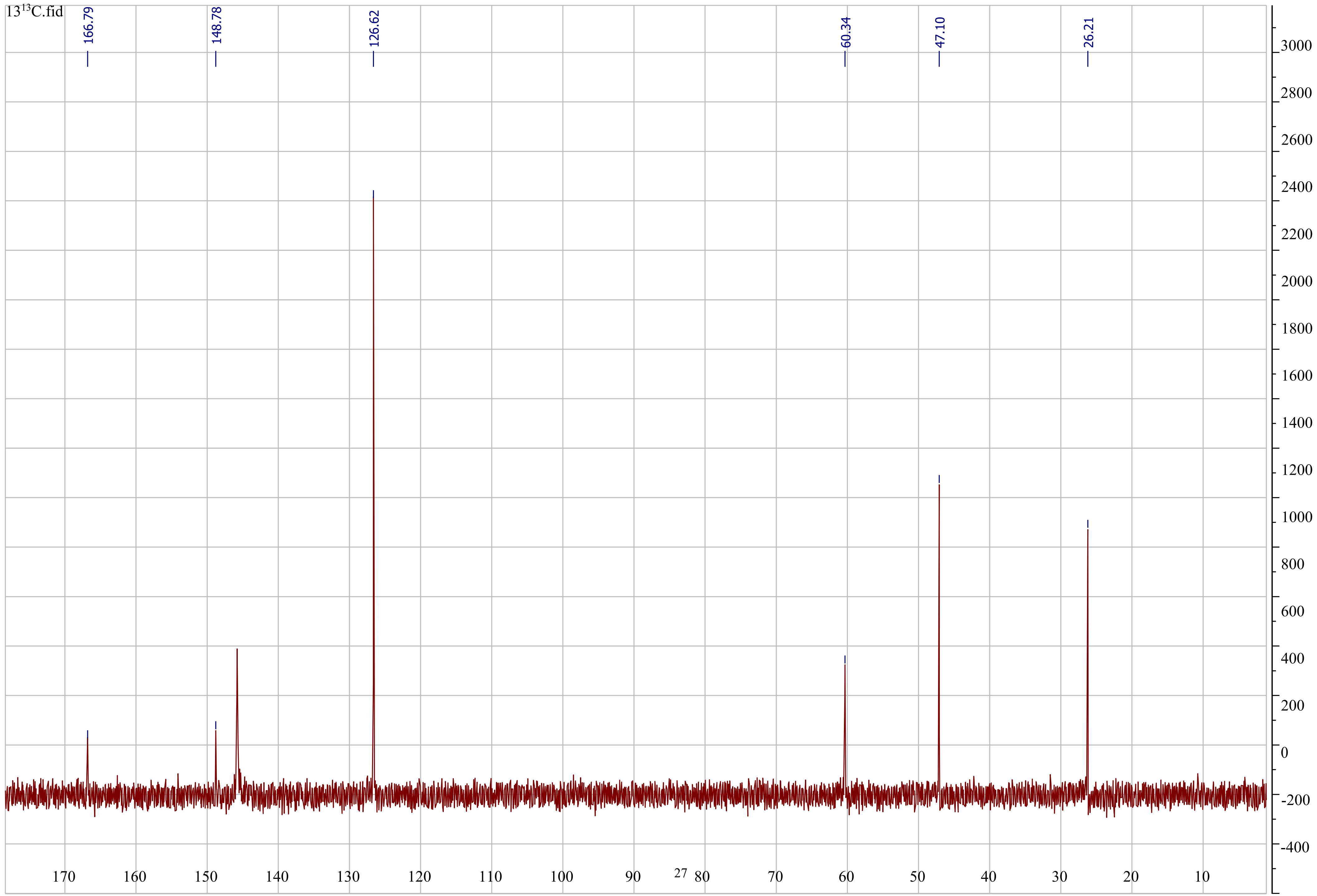
13 13C.fid
166.79
148.78
126.62
60.34
47.10
26.21
3000
2800
2600
2400
2200
2000
1800
1600
1400
1200
1000
800
600
400
200
0
-200
-400
170
160
150
140
130
120
110
100
90
80
70
60
50
40
30
20
10

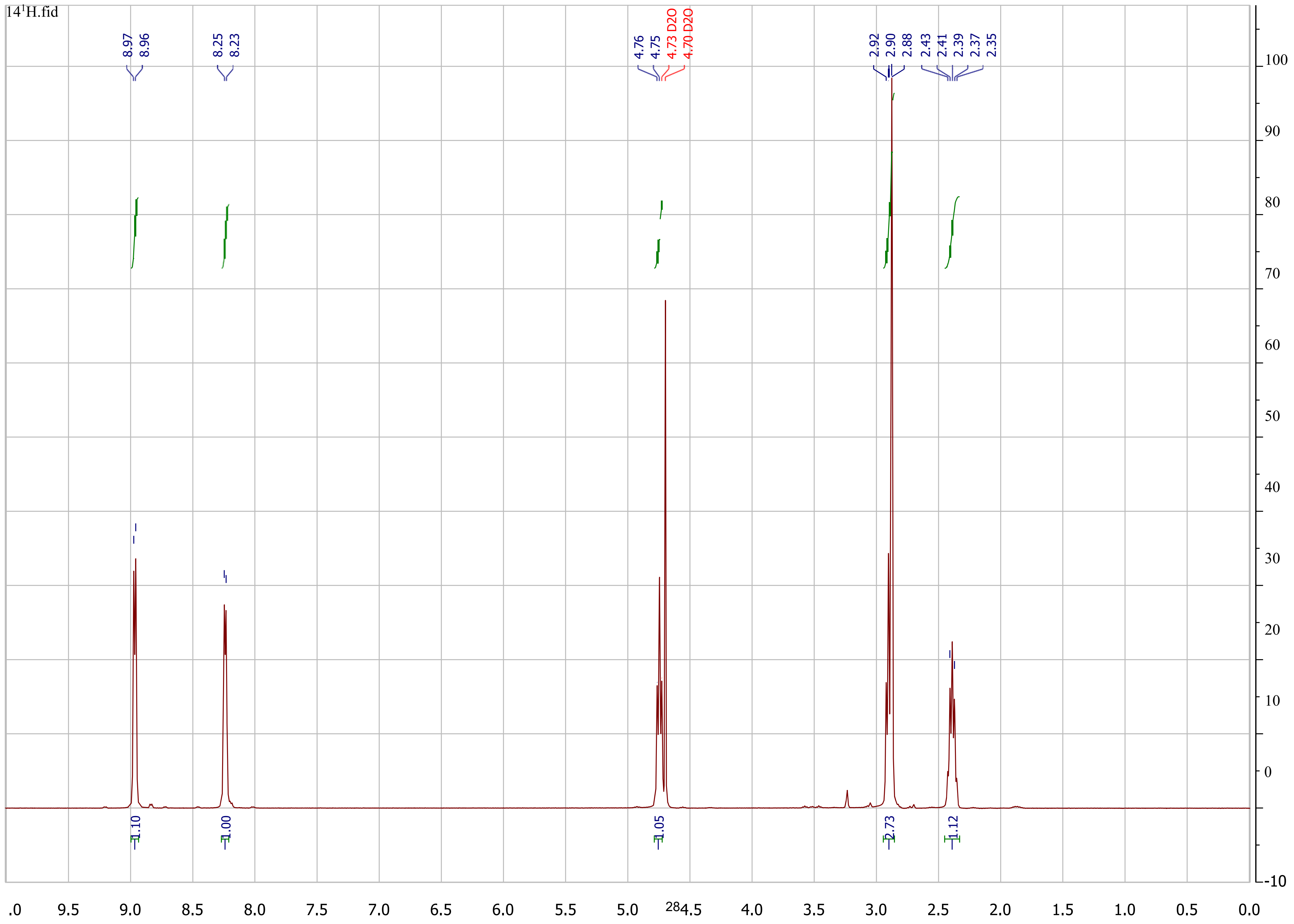

14¹H.fid
8.97
8.96
8.25
8.23
4.76
4.75
4.73 D2O
4.70 D2O
2.92
2.90
2.88
2.43
2.41
2.39
2.37
2.35
1.10
1.00
1.05
2.73
1.12

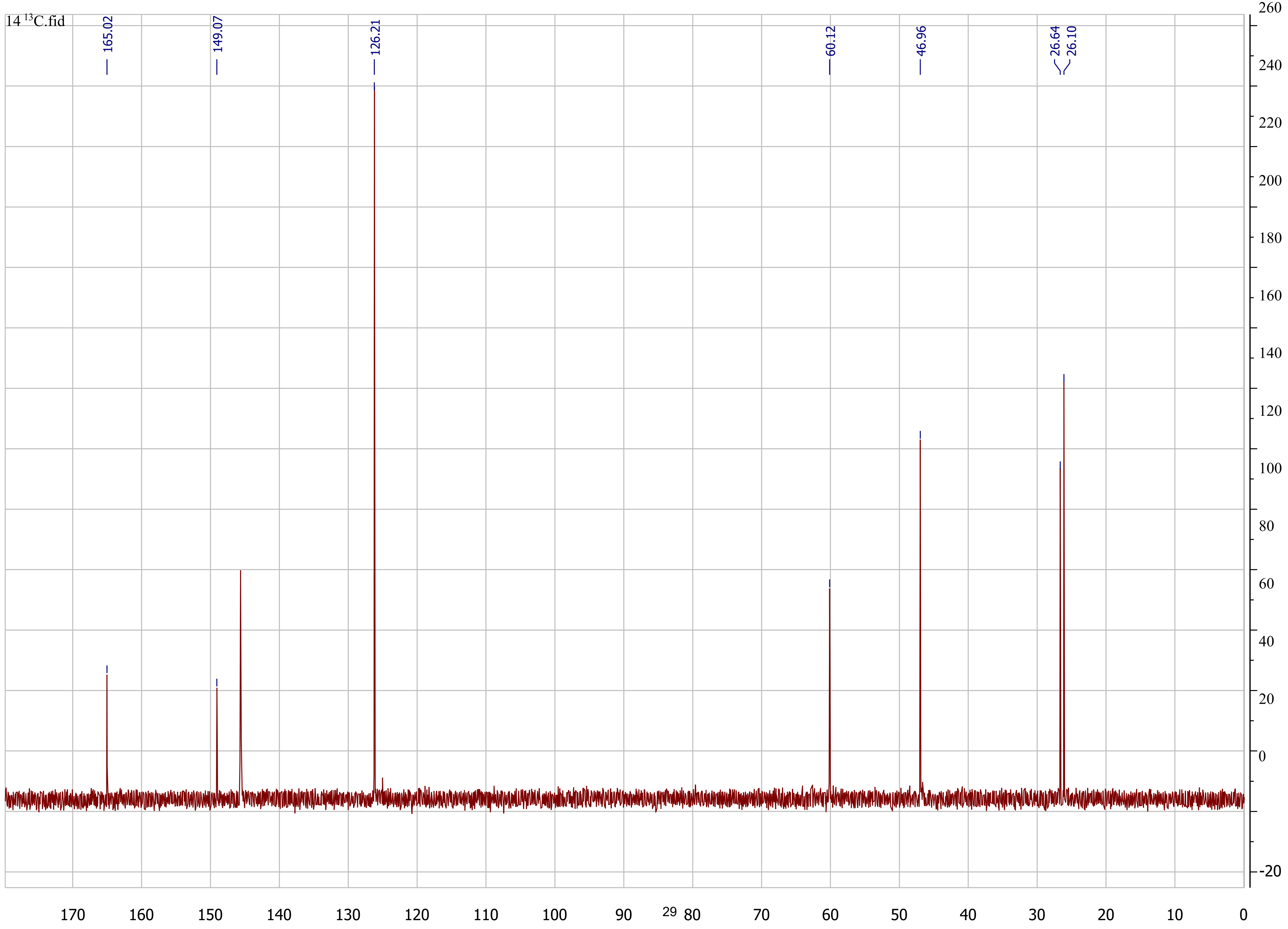

14 13C.fid
165.02
149.07
126.21
60.12
46.96
26.64
26.10
170
160
150
140
130
120
110
100
90
80
70
60
50
40
30
20
10
0
260
240
220
200
180
160
140
120
100
80
60
40
20
0
-20

29

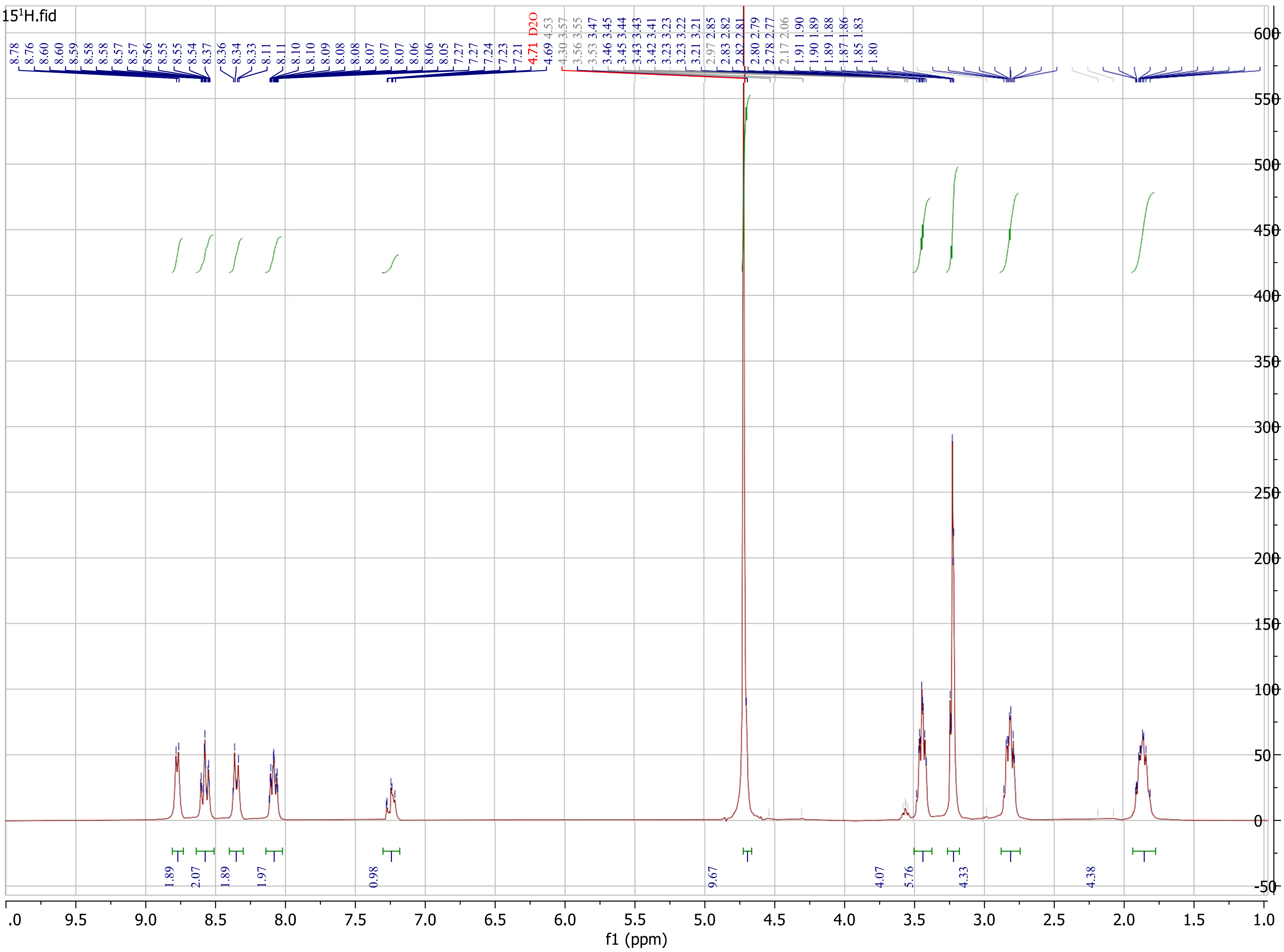
15¹H.fid
8.78 8.76 8.60 8.60 8.59 8.58 8.58 8.57 8.57 8.56 8.55 8.55 8.54 8.37 8.36 8.34 8.33 8.11 8.11 8.10 8.10 8.09 8.08 8.08 8.07 8.07 8.07 8.06 8.06 8.05 7.27 7.27 7.24 7.23 7.21 4.71 D2O 4.69 4.53 4.30 3.57 3.56 3.55 3.53 3.47 3.46 3.45 3.45 3.44 3.43 3.43 3.42 3.41 3.23 3.23 3.23 3.22 3.21 3.21 2.97 2.85 2.83 2.82 2.82 2.81 2.80 2.79 2.78 2.77 2.17 2.06 1.91 1.90 1.90 1.89 1.89 1.88 1.87 1.86 1.85 1.83 1.80
1.89
2.07
1.89
1.97
0.98
9.67
4.07
5.76
4.33
4.38
f1 (ppm)

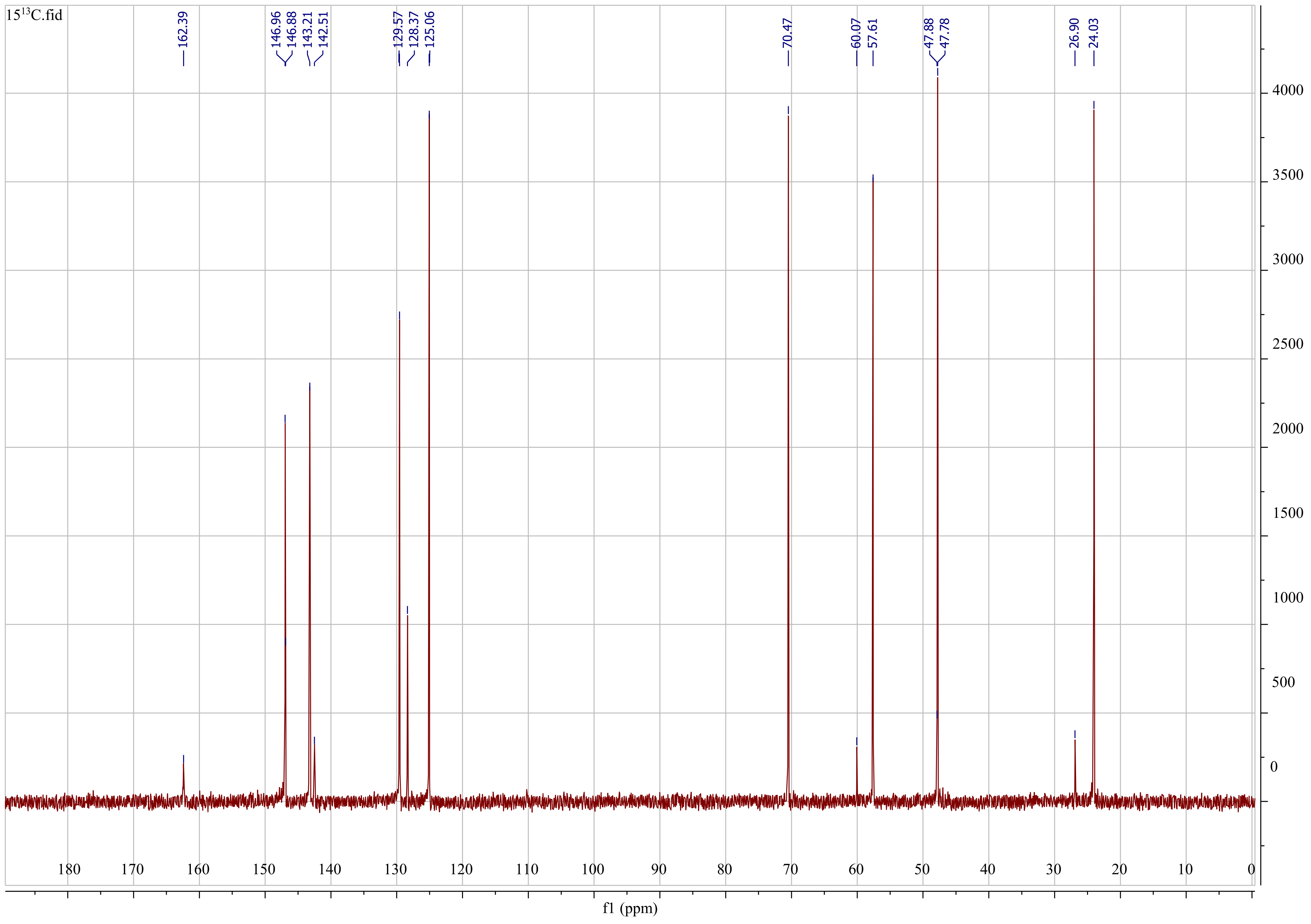
15[13]C.fid
162.39
146.96
146.88
143.21
142.51
129.57
128.37
125.06
70.47
60.07
57.61
47.88
47.78
26.90
24.03
4000
3500
3000
2500
2000
1500
1000
500
0
180 170 160 150 140 130 120 110 100 90 80 70 60 50 40 30 20 10 0
f1 (ppm)

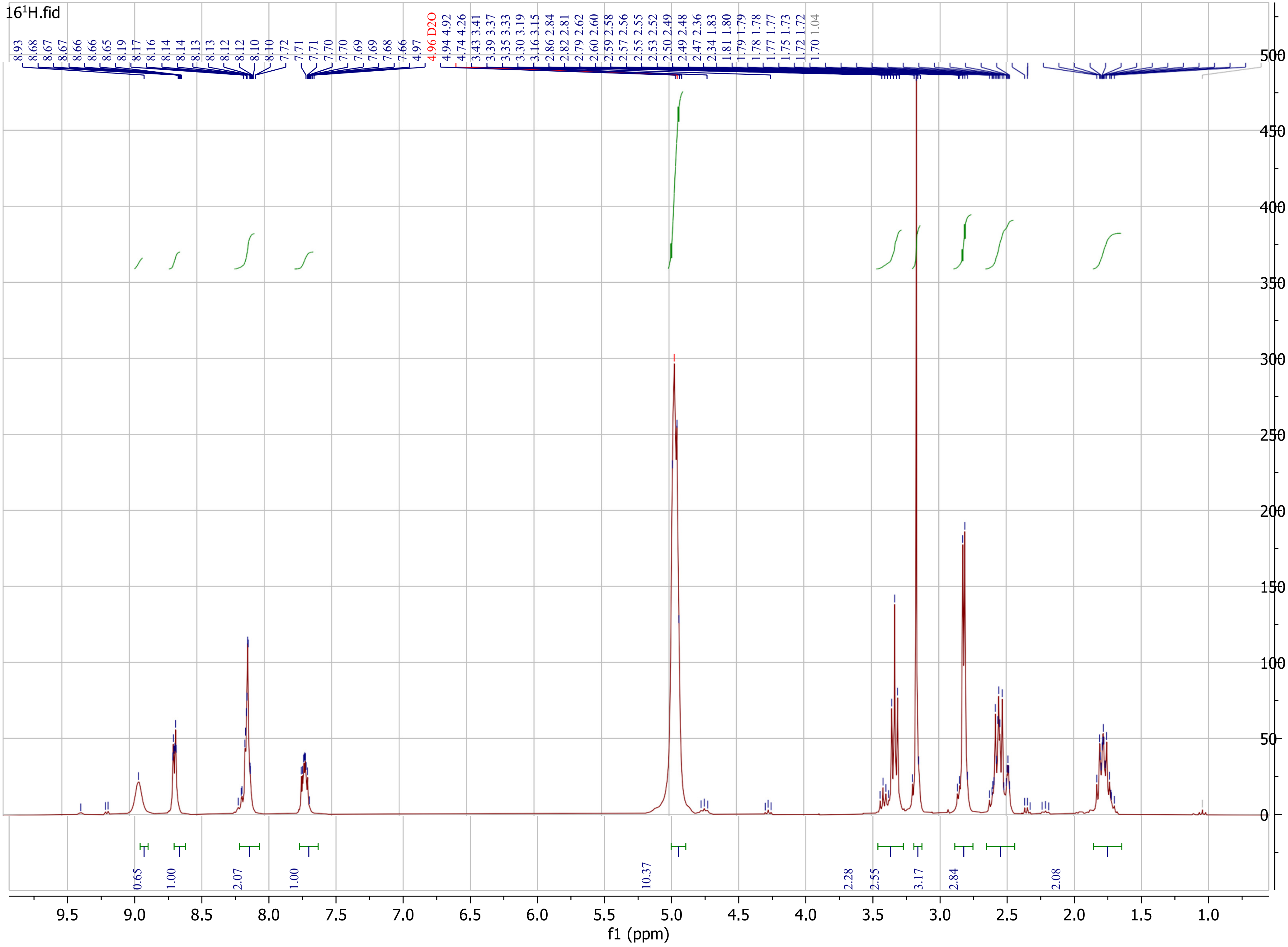

16¹H.fid
8.93 8.68 8.67 8.67 8.66 8.66 8.65 8.19 8.17 8.16 8.14 8.14 8.13 8.13 8.12 8.12 8.10 8.10 7.72 7.71 7.71 7.70 7.70 7.69 7.69 7.68 7.66 4.97
4.96 D2O
4.94 4.92 4.74 4.26 3.43 3.41 3.39 3.37 3.35 3.33 3.30 3.19 3.16 3.15 2.86 2.84 2.82 2.81 2.79 2.62 2.60 2.60 2.59 2.58 2.57 2.56 2.55 2.55 2.53 2.52 2.50 2.49 2.49 2.48 2.47 2.36 2.34 1.83 1.81 1.80 1.79 1.79 1.78 1.78 1.77 1.77 1.75 1.73 1.72 1.72 1.70 1.04
0.65 1.00 2.07 1.00 10.37 2.28 2.55 3.17 2.84 2.08
9.5 9.0 8.5 8.0 7.5 7.0 6.5 6.0 5.5 5.0 4.5 4.0 3.5 3.0 2.5 2.0 1.5 1.0
f1 (ppm)
0 50 100 150 200 250 300 350 400 450 500

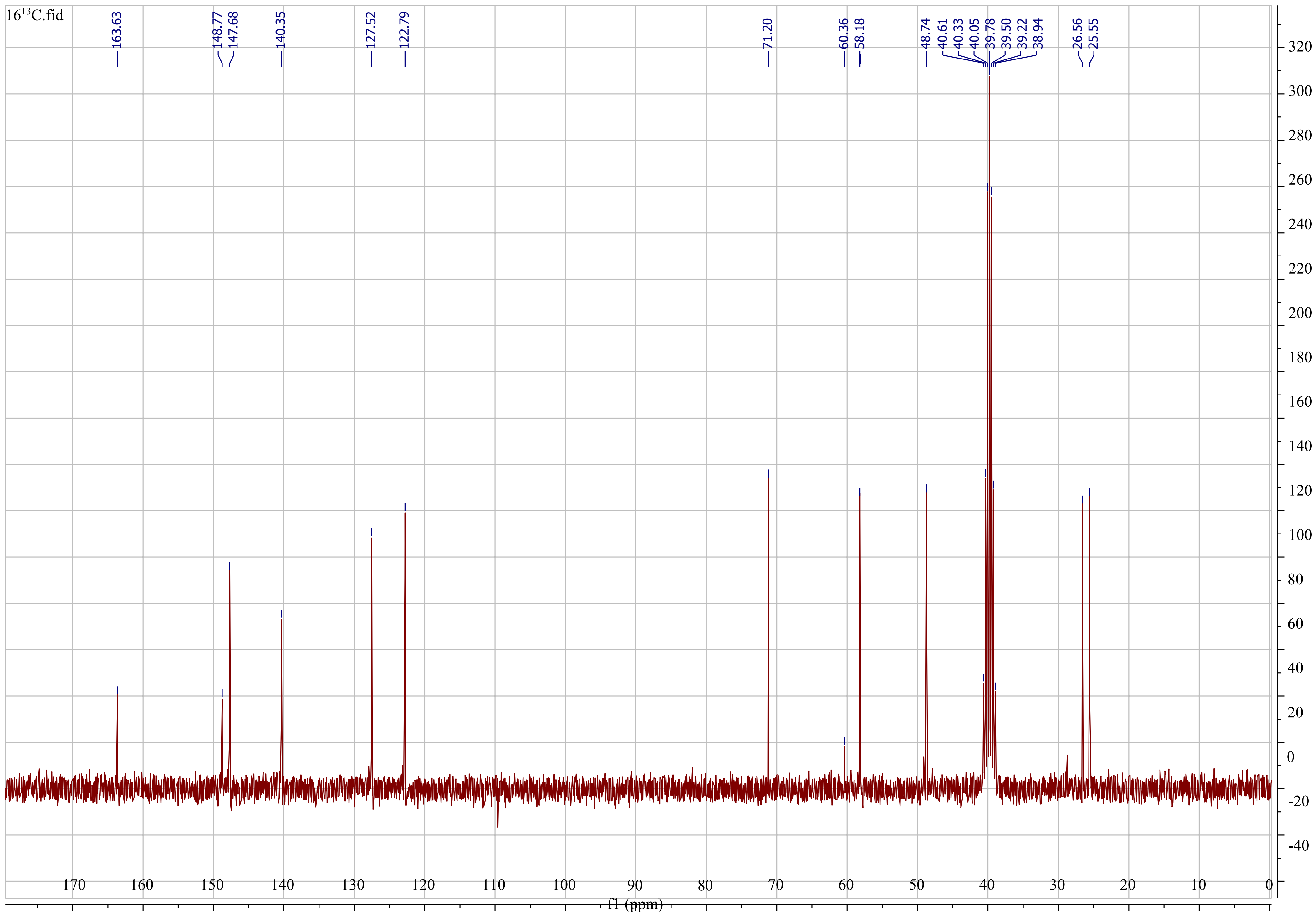
16 13C.fid
163.63
148.77
147.68
140.35
127.52
122.79
71.20
60.36
58.18
48.74
40.61
40.33
40.05
39.78
39.50
39.22
38.94
26.56
25.55
f1 (ppm)

Display Report

Analysis Info

Analysis Name	D:\Data\Burykina\8-22.d	Acquisition Date	30.06.2022 12:18:32
Method	tune_100-1200.m	Operator	BDAL@DE
Sample Name	16	Instrument / Ser#	maXis 43
Comment			

Acquisition Parameter

Source Type	ESI	Ion Polarity	Positive	Set Nebulizer	1.0 Bar
Focus	Active			Set Dry Heater	200 °C
Scan Begin	50 m/z	Set Capillary	4500 V	Set Dry Gas Set	4.0 l/min
Scan End	1800 m/z	Set End Plate Offset	-500 V	Divert Valve	Waste

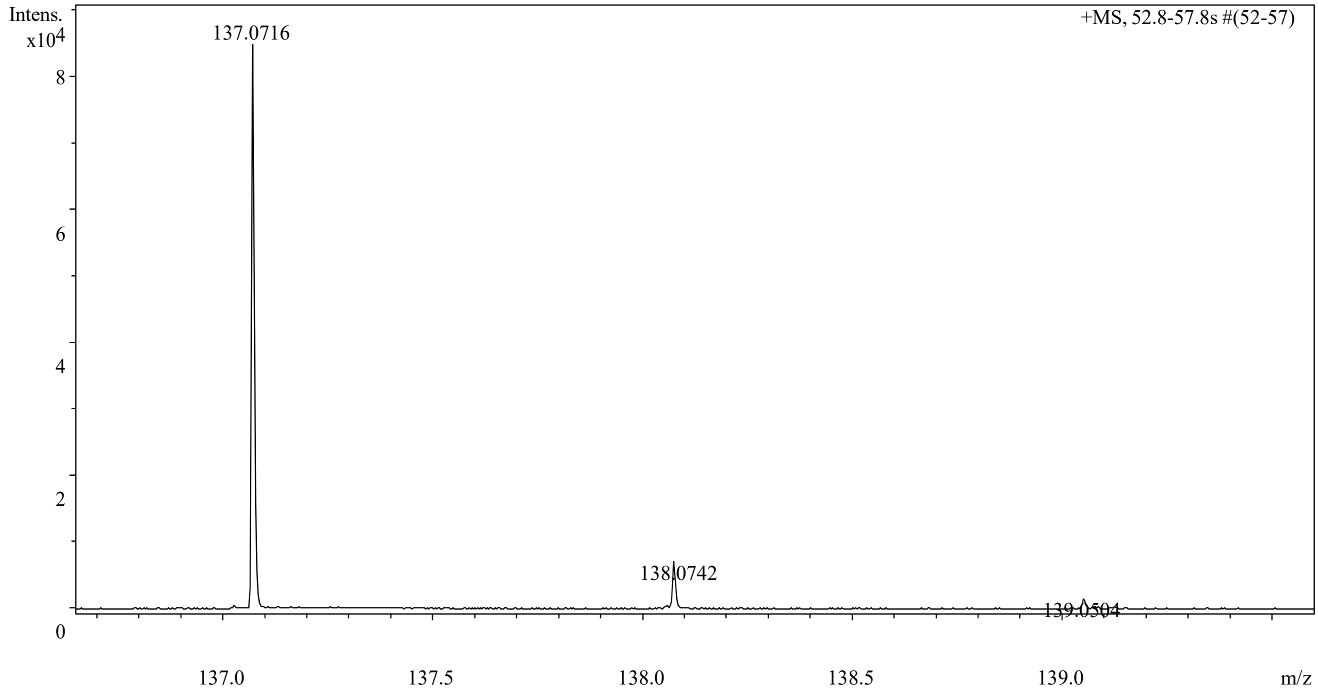

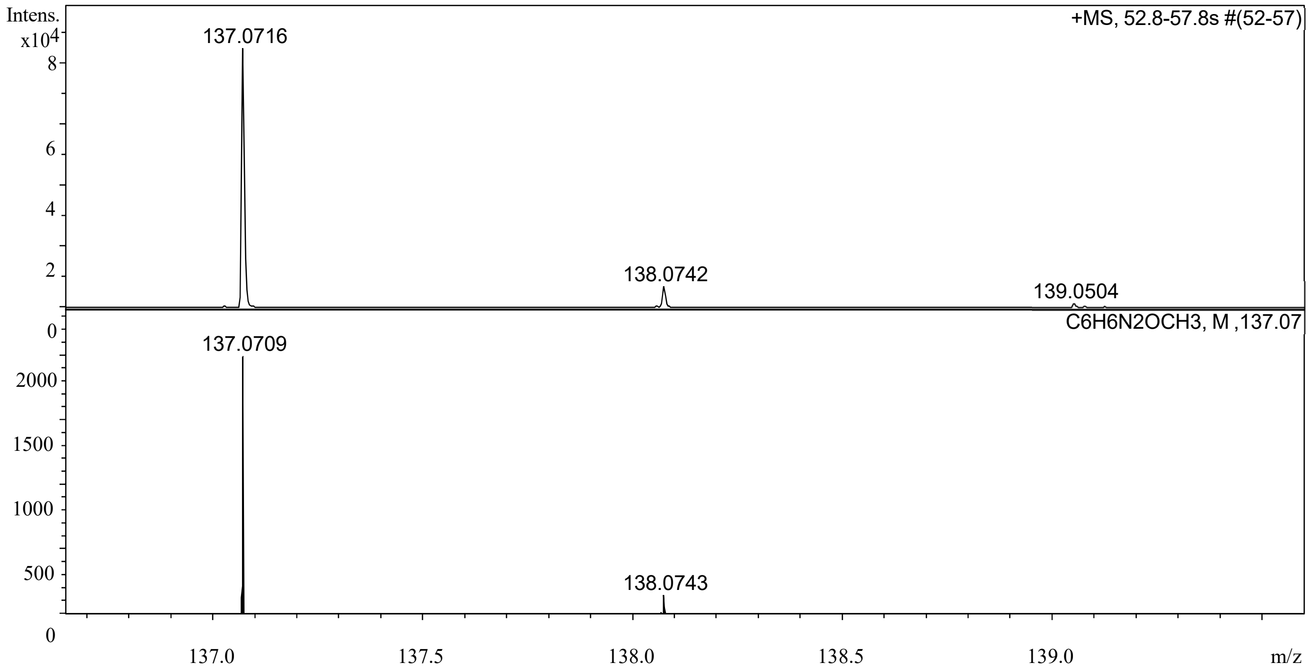

Display Report

Analysis Info

Analysis Name	D:\Data\Burykina\8-22neg.d	Acquisition Date	30.06.2022 12:29:57
Method	tune_100-1200.m	Operator	BDAL@DE
Sample Name	16	Instrument / Ser#	maXis 43
Comment			

Acquisition Parameter

Source Type	ESI	Ion Polarity	Negative	Set Nebulizer	1.0 Bar
Focus	Active			Set Dry Heater	200 °C
Scan Begin	50 m/z	Set Capillary	4000 V	Set Dry Gas Set	4.0 l/min
Scan End	1800 m/z	Set End Plate Offset	-500 V	Divert Valve	Waste

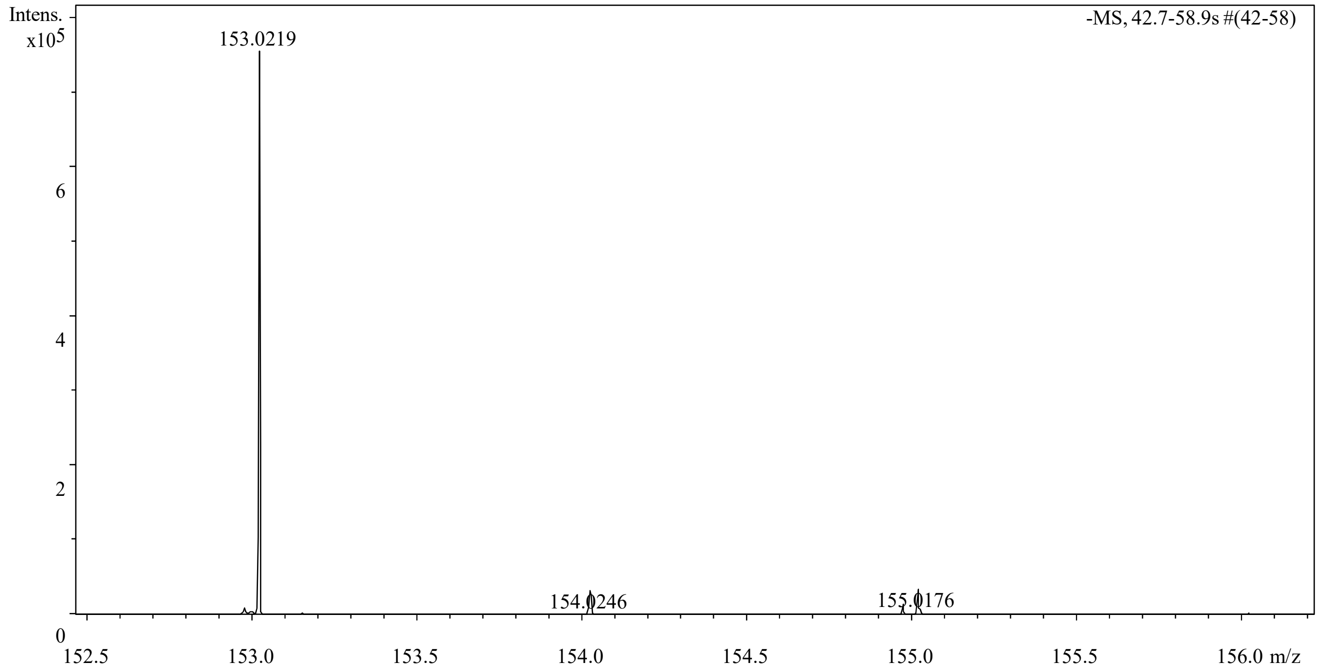

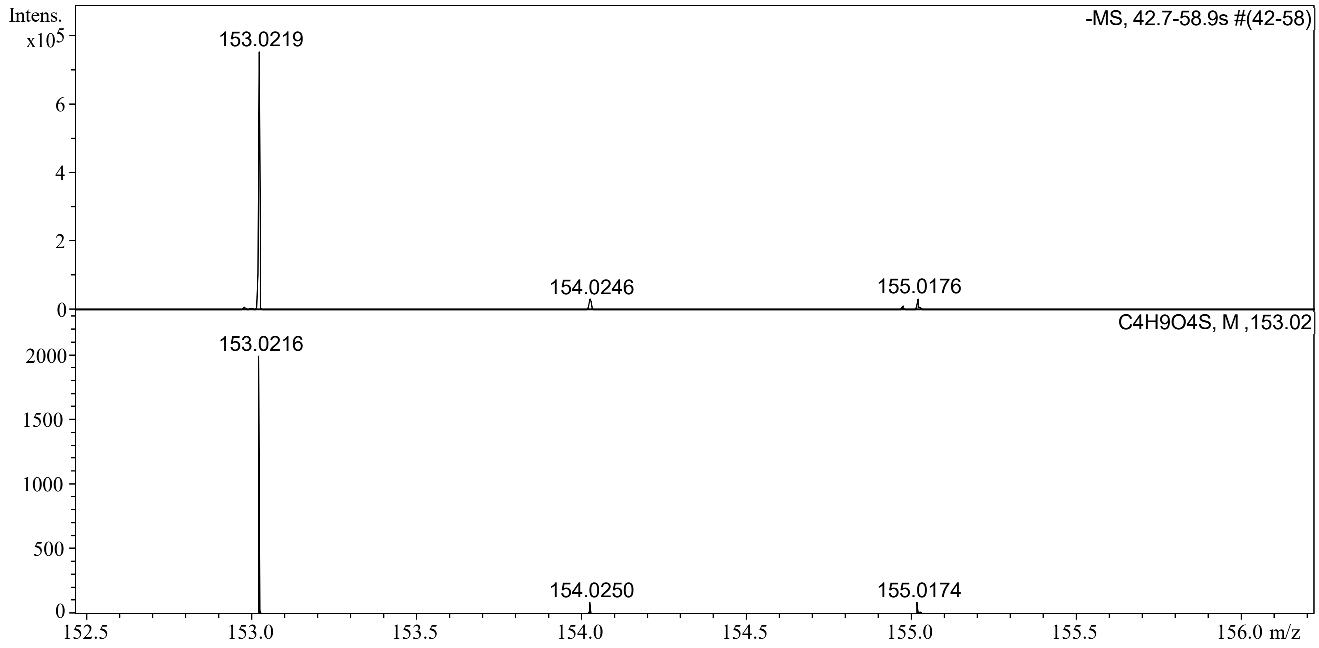

Figure S5 Effect of compounds **8-13** on the expression level of IL-1β, COX-2, and iNOS mRNA in LPS-activated Raw 264.7 cells. Raw 264.7 cells were pretreated with LPS (10 µg/mL) for 18 h followed by addition of Compounds **8-13** at concentrations of 0.001, 0.01, 0.1 and 0.5 mg/mL for 6 h. mRNA levels were analyzed with real-time PCR. The results are the relative levels of mRNA expression, which were normalized relative to the reference gene (Rpl27). Data are presented as mean and SD (n = 3).

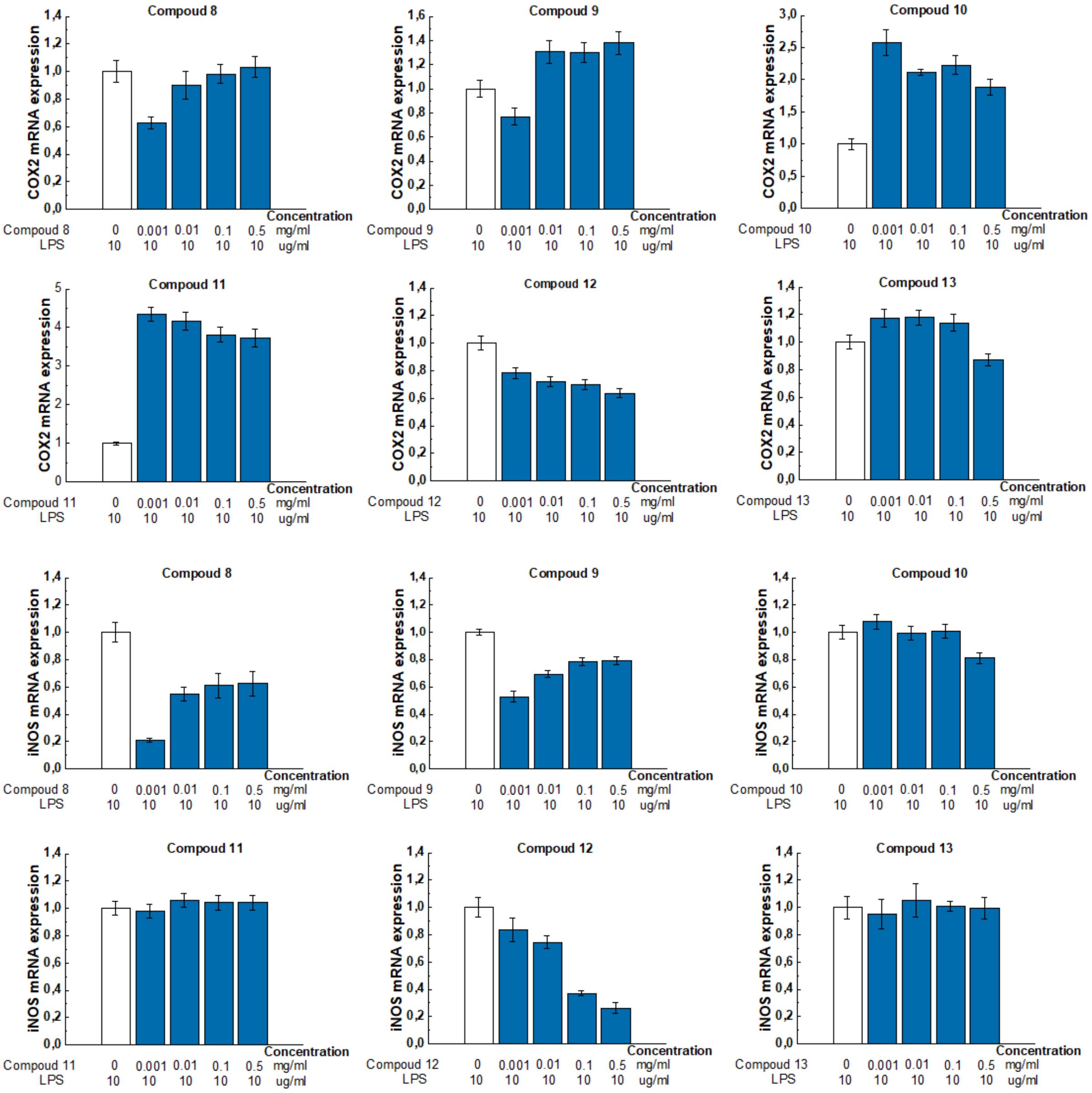

Supplement: Supplementary file 1 [file molecules-27-07542-s001.zip › molecules-1964220-SI.pdf]
